# Gain and isolation enhancement of a wideband MIMO antenna using metasurface for 5G sub-6 GHz communication systems

**DOI:** 10.1038/s41598-022-13522-5

**Published:** 2022-06-08

**Authors:** Md. Mhedi Hasan, Mohammad Tariqul Islam, Md Samsuzzaman, Mohd Hafiz Baharuddin, Mohamed S. Soliman, Ahmed Alzamil, Iman I. M. Abu Sulayman, Md. Shabiul Islam

**Affiliations:** 1 Department of Electrical, Electronic and Systems Engineering, Faculty of Engineering and Built Environment, Universiti Kebangsaan Malaysia, 43600 Bangi, Malaysia; 2 Department of Information and Communication Technology (ICT), Faculty of Engineering, Comilla University, Cumilla, 3506 Bangladesh; 3 Electrical Engineering Department, College of Engineering, University of Ha’il, Ha’il, 81481 Saudi Arabia; 4 Department of Computer and Communication Engineering, Faculty of Computer Science and Engineering, Patuakhali Science and Technology University, Patuakhali, Bangladesh; 5 Department of Electrical Engineering, College of Engineering, Taif University, PO Box 11099, Taif, 21944 Saudi Arabia; 6 Faculty of Engineering (FOE), Multimedia University, Persiaran Multimedia, 63100 Cyberjaya, Selangor Malaysia

**Keywords:** Metamaterials, Electrical and electronic engineering

## Abstract

This work proposes a compact metasurface (MS)-integrated wideband multiple-input multiple-output (MIMO) antenna for fifth generation (5G) sub-6 GHz wireless communication systems. The perceptible novelty of the proposed MIMO system is its wide operating bandwidth, high gain, lower interelement gap, and excellent isolation within the MIMO components. The radiating patch of the antenna is truncated diagonally with a partially ground plane, and a metasurface has been employed for enhancing the antenna performance. The suggested MS integrated single antenna prototype has a miniature dimension of 0.58λ × 0.58λ × 0.02λ. The simulated and measured findings demonstrate a wideband characteristic starting from 3.11 to 7.67 GHz including a high realized gain of 8 dBi. The four-element MIMO system has been designed by rendering each single antenna orthogonally to one another while retaining compact size and wideband properties between 3.2 and 7.6 GHz. The suggested MIMO prototype has been designed and fabricated on a low loss Rogers RT5880 substrate with a miniature dimension of 1.05λ × 1.05λ × 0.02λ and its performance is evaluated using a suggested 10 × 10 array of a square enclosed circular split ring resonators within the same substrate material. The inclusion of the proposed metasurface with a backplane significantly reduces antenna backward radiation and manipulates the electromagnetic field, thus improving the bandwidth, gain and isolation of MIMO components. The suggested 4-port MIMO antenna offers a high realized gain of 8.3 dBi compared to existing MIMO antennas with an excellent average total efficiency of 82% in the 5G sub-6 GHz spectrum and is in good accordance with measured results. Furthermore, the developed MIMO antenna exhibits outstanding diversity characteristics in respect of envelope correlation coefficient (ECC) less than 0.004, diversity gain (DG) close to 10 dB (> 9.98 dB) and high isolation between MIMO components (> 15.5 dB). Therefore, the proposed MS-inspired MIMO antenna substantiates its applicability for 5G sub-6 GHz communication networks.

## Introduction

**Figure 1:**
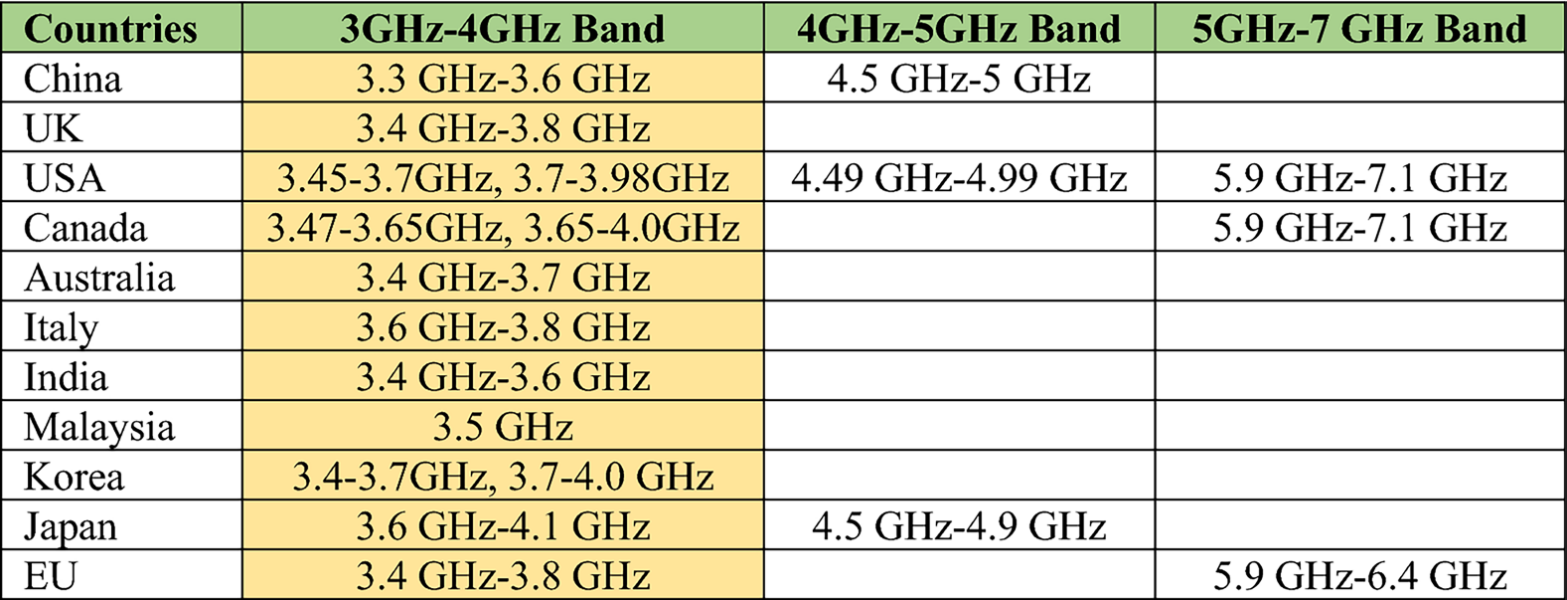
The worldwide spectrum allocation for 5G sub-6 GHz applications.

5G technology is an incredible advancement in wireless connectivity that enables faster and more secure networks for billions of connected devices with "zero" latency user experiences (delay less than 1 ms) and introduces emerging services including e-Health, smart education, smart cities, smart homes, virtual reality (VR), smart factories, and the Internet of Vehicles (IoV), transforming our lives, society, and industries^1–3^. 5G spectrum is divided into four bands by the Federal Communications Commission (FCC)^4^. The sub-6 GHz frequency band piqued the researchers' interest since it permits long-distance communication at a high data rate^5,6^. The allotment of the 5G sub-6 GHz spectrum for global 5G connectivity is visualized in Fig. 1, indicating that all nations are contemplating the sub-6 GHz for 5G communications^7,8^. The antenna is a crucial component of the 5G network, and more base stations and user terminal antennas will be required.

The microstrip patch antenna offers the advantages of a low profile and planar construction but suffers from limited bandwidth and gain^9,10^; consequently, much research has been conducted to increase the antenna's gain and bandwidth. Metasurface (MS) has been extensively employed in antenna technology in recent years, particularly for increasing gain and bandwidth^11,12^; however, these antennas are restricted to a single port. MIMO technology is a crucial aspect of wireless communications because it enables the simultaneous use of multiple antennas for data traffic, thereby increasing the data transfer rate, spectral efficiency, channel capacity, and reliability^13–15^. MIMO antennas are a potential candidate for 5G applications because they enable data transmission and reception over multiple channels without requiring extra power^16,17^. The mutual coupling effect between MIMO components, which is dependent on the location of the MIMO elements and the gain of the MIMO antenna are major issues among researchers. Various MIMO antennas operating in the 5G sub-6 GHz spectrum are presented in ^18–20^, all of which exhibited good isolation and MIMO performance. However, the gain and operational bandwidth of these proposed systems are lower.

Metamaterial (MM) is a novel material that does not exist in nature and can manipulate electromagnetic waves, therefore improving the antenna's performance^21–24^. MM is currently widely used in antenna technology to improve radiation pattern, bandwidth, gain, and isolation among antenna elements and wireless communication systems, as stated in^25–28^. Metasurface-based four-elements MIMO system is devised in^29^, where the antenna patch is sandwiched between a metasurface and the ground without an air gap, improving the MIMO performance. However, this design has large dimensions and a lower operating frequency, as well as a complicated design structure. Electromagnetic bandgap (EBG) and ground stub were incorporated into the proposed 2-port wideband MIMO antenna to increase the isolation of MIMO components^30^. The designed antenna offers good MIMO diversity performance and excellent isolation between two MIMO antennas but only has two MIMO components with low gain. Also, in^31^, an ultra-wideband (UWB) two-port MIMO antenna is presented, whose MIMO performance is explored using metamaterial. While this antenna is capable of operating at UWB, it has a low gain and poor isolation between two antennas. The work in^32^ proposed a 2-port MIMO system with an electromagnetic bandgap (EBG) reflector to improve the gain. Although the developed antenna arrays offer high gain and good MIMO diversity performance, however, it has a large size that hinders their applicability in the next generation communication gadgets. Another reflector-based wideband antenna is developed in^33^, where the reflector is integrated beneath the antenna with a large air gap of 22 mm, indicating a low peak gain of 4.87 dB. A four-port MIMO antenna has been developed for millimetre-wave applications in paper^34^, which is integrated with an MS layer to improve the isolation and gain of the MIMO system. However, this antenna offers good gain and isolation but has limited bandwidth and poor mechanical performance due to the wide air gap. Similarly, a three-pair metasurface integrated bow-tie-shaped 4-port MIMO antenna with the highest gain of 7.4 dBi is devised in^35^ for mm-wave communications. In^36^, the MS has been utilized on the backside of the 5G antenna for improving the antenna gain, where the metasurface act as a reflector. However, the MS structure is asymmetric, and the unit cell structure has received less attention.

According to the findings of the above analysis, none of the stated antennas has high gain with excellent isolation and MIMO characteristics, as well as wideband coverage. As a consequence, a metasurface-based MIMO antenna that covers a wide frequency range in the 5G sub-6 GHz spectrum with high gain and isolation is still required. Keeping in view the constraints of the above literature, a metasurface-based wideband, high gain four-element MIMO antenna system with outstanding diversity qualities is suggested for 5G sub-6 GHz wireless communication systems. Moreover, the suggested MIMO antenna exhibits outstanding isolation among the MIMO components, low interelement gap, and high radiation efficiency. The antenna patch is truncated diagonally and positioned on top of the proposed metasurface with a 12 mm air gap, reflecting the antenna back radiation and improving the antenna gain and directivity. Furthermore, the suggested single antenna is used to design a four-element MIMO antenna with outstanding MIMO performance by positioning each antenna orthogonal to one another. The developed MIMO antenna is then integrated on top of the 10 × 10 array of MS with a copper backplane to enhance the radiation characteristics. This design is distinguished by its wide operating range (3.08–7.75 GHz), high gain of 8.3 dBi and high average total efficiency of 82%, as well as excellent isolation of greater than − 15.5 dB between MIMO antenna components. The 3D electromagnetic software CST Studio suite 2019 was used to simulate the developed MS-based MIMO antenna and confirmed through the experimental investigation.

## Single antenna design and analysis

This section goes through the proposed single antenna architecture and design approach in depth. Additionally, the simulated and observed findings are discussed in detail, including scattering parameters, gain, and total efficiency, with and without the usage of a metasurface. The antenna prototype is developed on a 1.575 mm thick low loss Rogers 5880 dielectric substrate material with a low dielectric constant value of 2.2. The electromagnetic simulator CST studio package 2019 is used to develop and simulate this design.

### Single antenna geometry and design procedure

**Figure 2:**
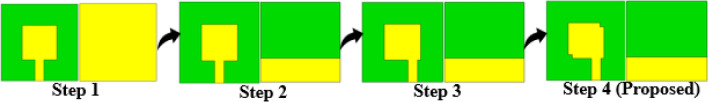
The design evolution of the single proposed antenna. (CST STUDIO SUITE 2019).

**Figure 3:**
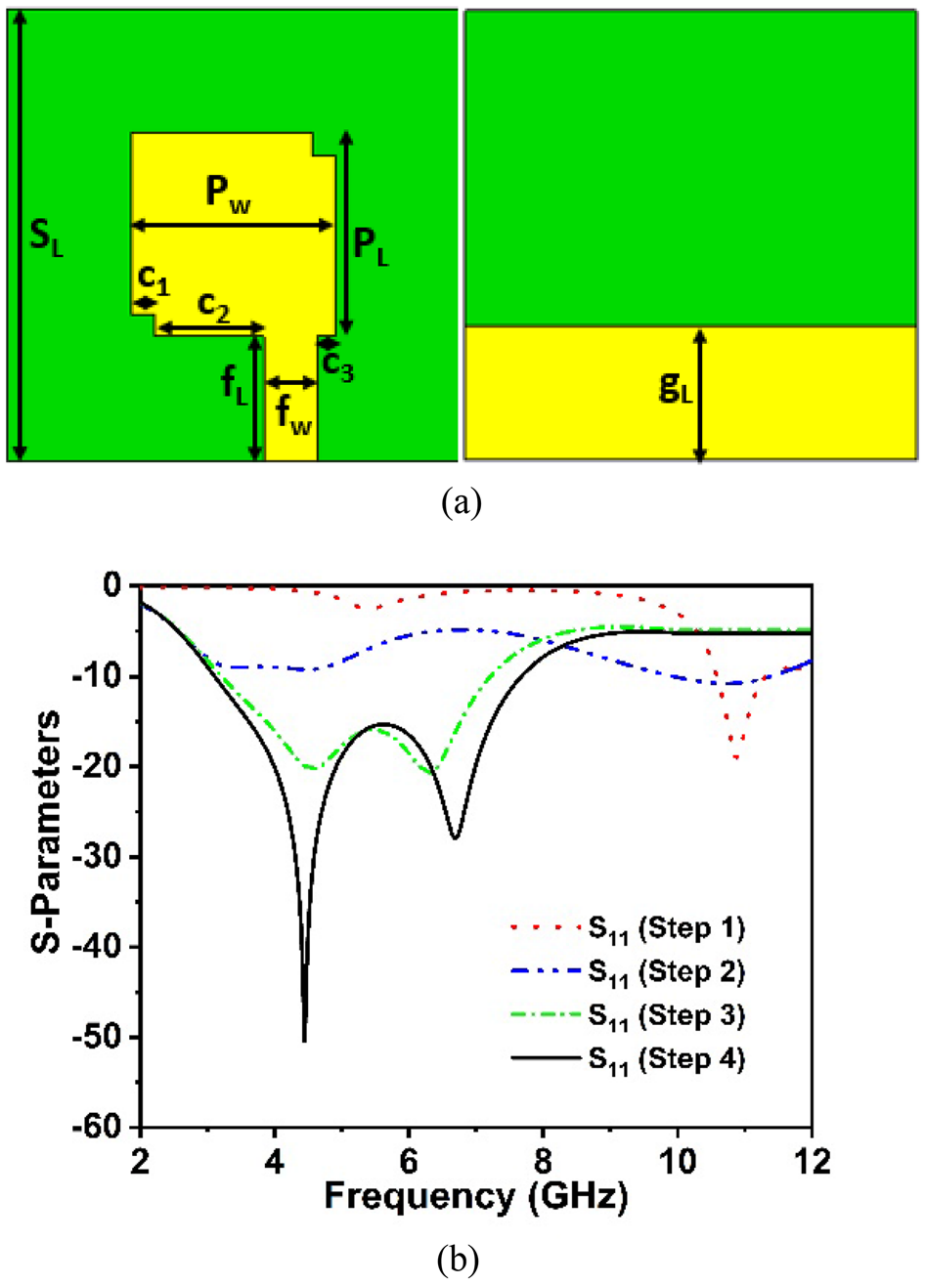
(**a**) Top and back view of the designed single antenna (CST STUDIO SUITE 2019) (**b**) S-parameters curves.

Figure 2 depicts the suggested architecture and design modelling for a single-element antenna. According to the well-established mathematical formula^37^, the antenna comprises a square radiating patch with a line feed and a copper ground plane, as stated in step 1, and resonates at 10.8 GHz with a very narrow bandwidth, as seen in Fig. 3b. The initial antenna radiator patch dimension has been determined by the following mathematical relations^37^:

1 $$P_{w} = \frac{c}{{2f_{r} }}\sqrt {\frac{2}{{\gamma_{r} + 1}}}$$

2 $$P_{L} = \frac{c}{{2f_{r} \sqrt {\gamma_{reff} } }} - 2\Delta L$$

where $P_{L}$ and $P_{w}$ is the patch length and width, c denotes the velocity of light, $\gamma_{r}$ is the substrate dielectric constant, $\gamma_{reff}$ indicates the effective dielectric value of the radiating patch, and $\Delta L$ denotes the change in patch length. The antenna backplane is optimized in the second step, which increases impedance bandwidth, despite the very low − 10 dB value. In the third step, the position of the feed line is shifted to the right which improves the impedance bandwidth and impedance matching^38^ of the suggested antenna. The antenna exhibited excellent working bandwidth of 4 GHz during this phase while also covering the sub-6 GHz frequency spectrum in 5G. The fourth and final stage incorporates etching the square slots in the diagonal corners of the radiating patch. This slot expands the bandwidth of 4.56 GHz substantially, encompassing the 5G sub-6 GHz spectrum between 3.11 GHz and 7.67 GHz, as depicted in Fig. 3b. The front and bottom perspectives of the suggested design are indicated in Fig. 3a and the final optimized desired design parameters are as follows: *S*_*L*_ = 40 mm, *P*_*w*_ = 18 mm, *P*_*L*_ = 18 mm, *g*_*L*_ = 12 mm, *f*_*L*_ = 11 mm, *f*_*W*_ = 4.7 mm, *c*_1_ = 2 mm, *c*_2_ = 9.65 mm, and *c*_3_ = 1.65 mm.

## Metasurface design and characterization

**Figure 4:**
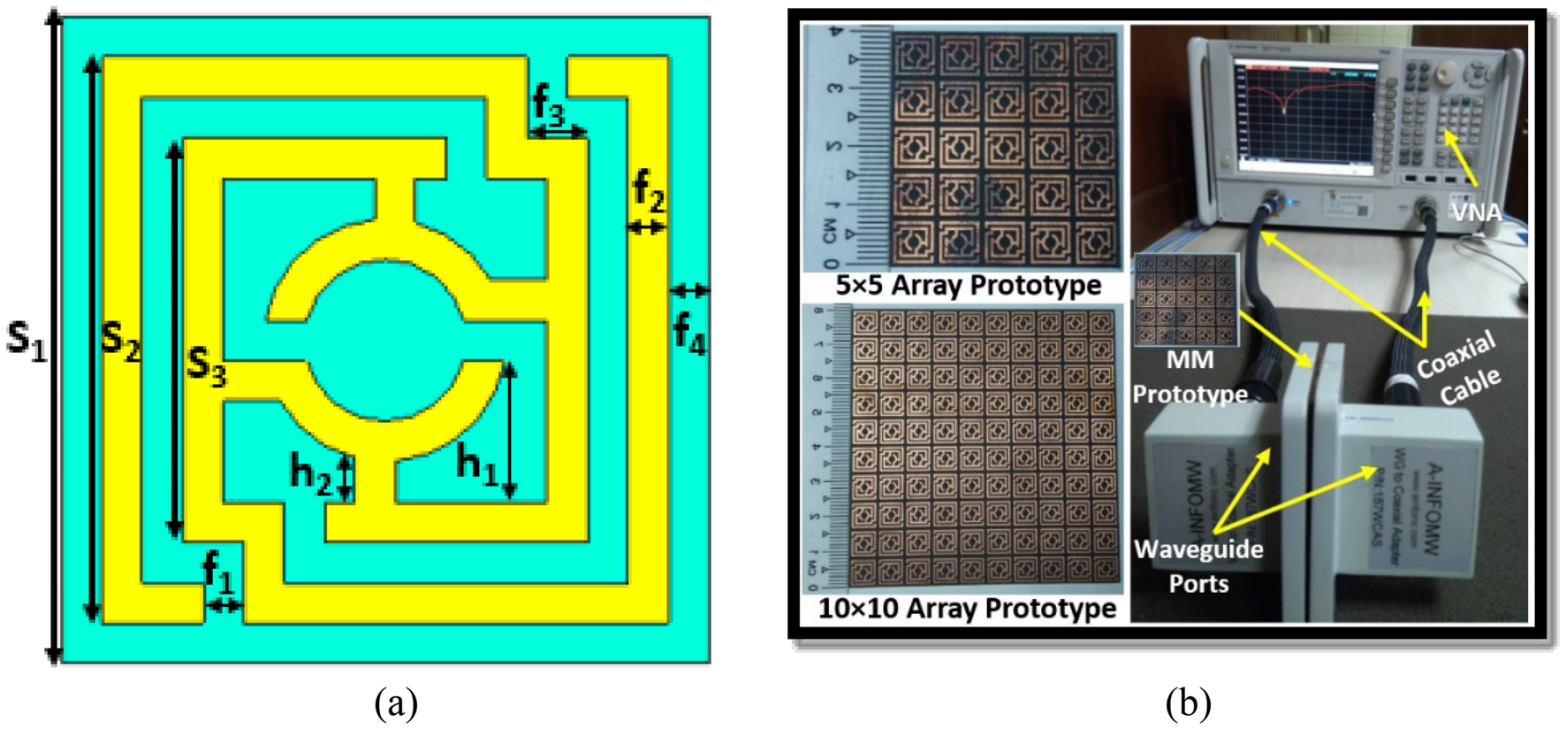
(**a**) Unit cell geometry (*S*_1_ = 8 mm, *S*_2_ = 7 mm, *S*_3_ = 5 mm, *f*_1_, *f*_2_, *f*_4_ = 0.5 mm, *f*_3_ = 0.75 mm, *h*_1_ = 0.5 mm, *h*_2_ = 1.75 mm) (CST STUDIO SUITE 2019) (**b**) Photographs of the MM measurement arrangement.

**Figure 5:**
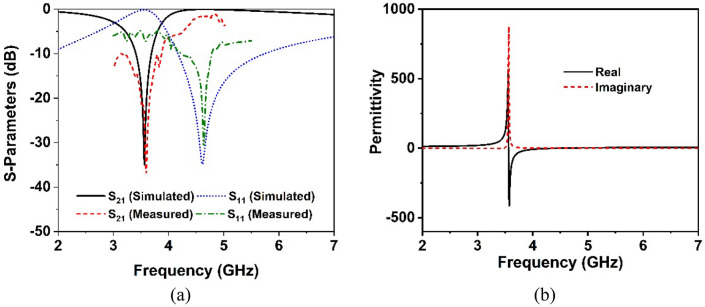
(**a**) Simulated and examined scattering parameter curves for the metamaterial prototype. (**b**) Permittivity curves for MM unit cell.

The metasurface is a term that refers to the periodic array of unit cells at a certain distance apart. The metasurface is a highly effective approach for improving the antenna's radiation performance, including bandwidth, gain and isolation between MIMO components. Due to the impact of surface wave propagation, the metasurface generates extra resonance, which contributes to the antenna's performance improvement^39^. An epsilon negative metamaterial (MM) unit cell has been proposed in this work operating at a 5G sub-6 GHz band. The MM with a surface area of 8mm × 8 mm has been developed on a low loss Rogers 5880 substrate with a dielectric constant of 2.2 and a thickness of 1.575mm. The optimized MM resonator patch is made up of an inner circular split ring that is coupled to the two modified outer split rings, as illustrated in Fig. 4a. Figure 4a summarizes the final optimized parameters for the proposed MM unit cell. Subsequently, 40 mm × 40 mm and 80 mm × 80 mm dimensions of metasurface layer without and with copper backplane have been developed by utilizing the 5 × 5 and 10 × 10 arrays of unit cells, respectively. The proposed MM structure is simulated utilizing the 3D electromagnetic simulation software “CST studio suite 2019”. The fabricated prototype of the suggested MM array structure and measurement setup (two-port PNA network analyzer and waveguide ports) are displayed in Fig. 4b in order to confirm the CST simulation findings by analyzing the real-world response. In the measurement setup, an Agilent PNA series network analyzer is utilized, coupled with two waveguide-to-coaxial adapters (A-INFOMW, P/N:187WCAS) for transmitting and receiving signals. The 5 × 5 array prototype is placed between the two waveguide-to-coaxial adapters which are connected via coaxial cable to the two-port network analyzer (Agilent PNA N5227A). The Agilent N4694-60001 calibration kit was used to calibrate the network analyzer in the experimental setup. The CST simulated and observed scattering parameters of the recommended MM array prototype are portrayed in Fig. 5a. It can be observed that the suggested MM structure is resonating in the 5G sub-6 GHz band. Despite a slight difference in the − 10 dB bandwidth, the simulated and experimental results are quite similar. The resonance frequency, bandwidth, and amplitude of the observed resonances differed slightly from that of the simulated ones, as seen in Fig. 5a. These disparities between observed and simulated findings are due to fabrication faults, a slight edge gap between prototype and waveguide ports, mutual coupling effects between waveguide ports and array components, and measurement tolerance. Moreover, the proper placement of the developed prototype between waveguide ports in the experimental setup leads to the shifting of resonances. In addition, unwanted noises were observed during the calibration phase, which contributed to the disparity between numerical and measured findings. However, beyond these difficulties, the suggested MM array prototype performs well, with a strong correlation between simulation and experiment, making it perfect for 5G sub-6 GHz wireless communication applications.

The effective relative parameters such as effective permittivity, permeability, and refractive index are studied using the CST electromagnetic simulator's built-in post-processing approach in order to further analyze the behavior of the MM unit cell. The MM effective parameters are deduced from the scattering parameters using a robust retrieval approach. The following transmission and reflection coefficient Eqs. (3) and (4) can be used to determine the refractive index and impedance (see reference^40^).

3 $${\text{Reflection}}\;{\text{coefficient}},\;S_{11} = \frac{{R_{01} \left( {1 - e^{{i2nk_{0} d}} } \right)}}{{1 - R^{2}_{01} e^{{i2nk_{0} d}} }}$$

4 $${\text{Transmission}}\;{\text{coefficient}},\;S_{21} = \frac{{\left( {1 - R^{2}_{01} } \right) e^{{ink_{0} d}} }}{{1 - R^{2}_{01} e^{{i2nk_{0} d}} }}$$

where $$R_{01} = \frac{z - 1}{{z + 1}}$$.

Calculate the impedance and refractive index by inverting Eqs. (3) and (4):

5 $${\text{Impedance}},\;z = \pm \sqrt {\frac{{\left( {1 + S_{11} } \right)^{2} - S^{2}_{21} }}{{\left( {1 - S_{11} } \right)^{2} - S^{2}_{21} }}}$$

6 $${\text{and}}\;{\text{the}}\;{\text{refractive}}\;{\text{index}},\;n = \frac{1}{{K_{0} d}} \left\{ {\left[ {\left[ {\ln \left( {e^{{ink_{0} d}} } \right)} \right]^{{\prime \prime }} + 2m\pi } \right] - i \left[ {\ln \left( {e^{{ink_{0} d}} } \right)} \right]^{{\prime }} } \right\}$$

**Figure 6:**
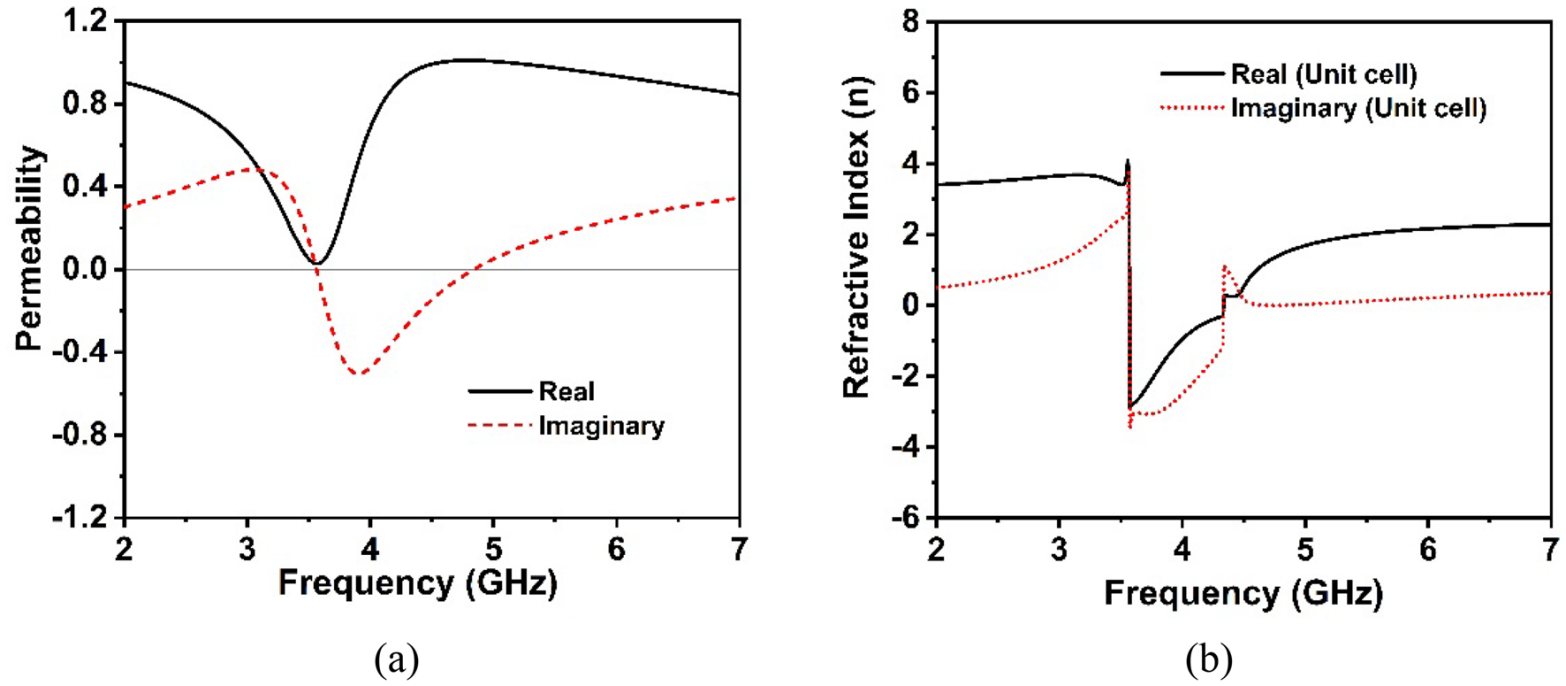
Extracted effective parameters (**a**) permeability (**b**) Refractive index of the suggested unit cell.

The real and imaginary parts of the operator are represented respectively by (.)′ and (.)″, whereas the integer value *m* corresponds to the real refractive index. Permittivity and permeability are determined using the formulae $\varepsilon { } = { }n/z,$ and $\mu = nz$, which are based on impedance and refractive index, respectively. The effective permittivity curves of the MM structure are depicted in Fig. 5b. At the resonance frequency, it is observed that the effective permittivity is negative. Figure 6a,b displays the extracted effective permeability (µ) values and effective refractive index (n) of the suggested unit cell. It is worth noticing that the extracted permeability exhibit near-zero positive real value, confirming that the recommended MM structure possesses epsilon negative (ENG) qualities. Furthermore, the resonances of near-zero permeability are strongly coupled in the resonance frequency, as demonstrated in Fig. 6a. The developed unit cell exhibits a negative refractive index (Fig. 6b), implying that this proposed MM could be used to enhance antenna performance^21,41^.

## Single antenna result analysis

**Figure 7:**
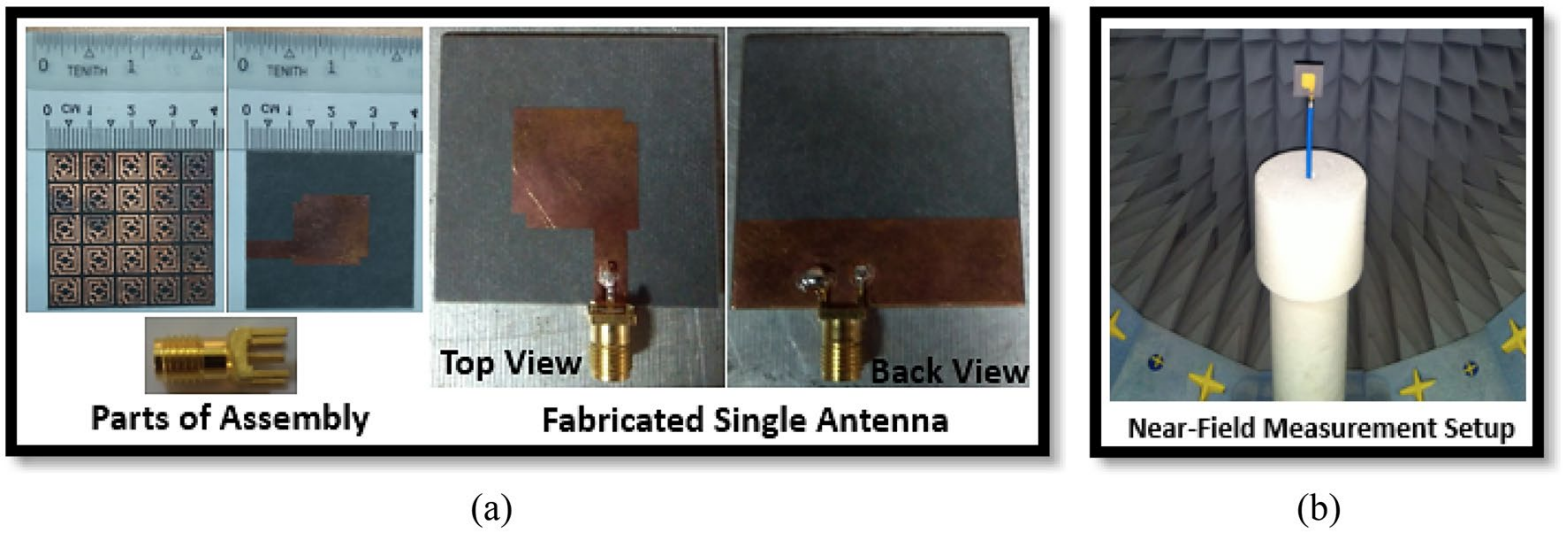
Photo shows (**a**) manufactured single antenna and its associated components. (**b**) Near-field (SATIMO) measurement arrangement.

**Figure 8:**
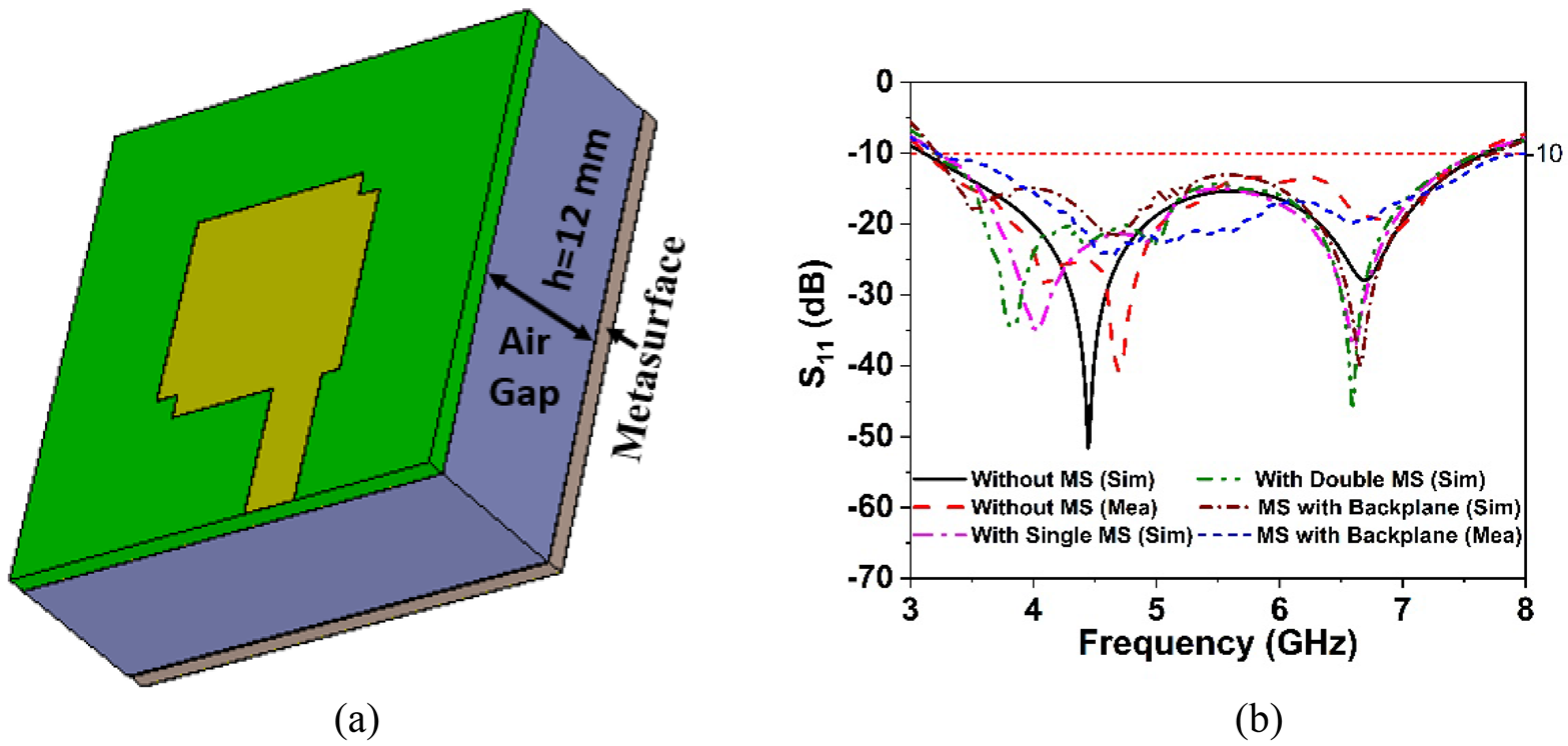
(**a**) Antenna excitation with metasurface reflector (CST STUDIO SUITE 2019). (**b**) Simulated and experimental reflection coefficient of the single antenna without and with MS.

**Figure 9:**
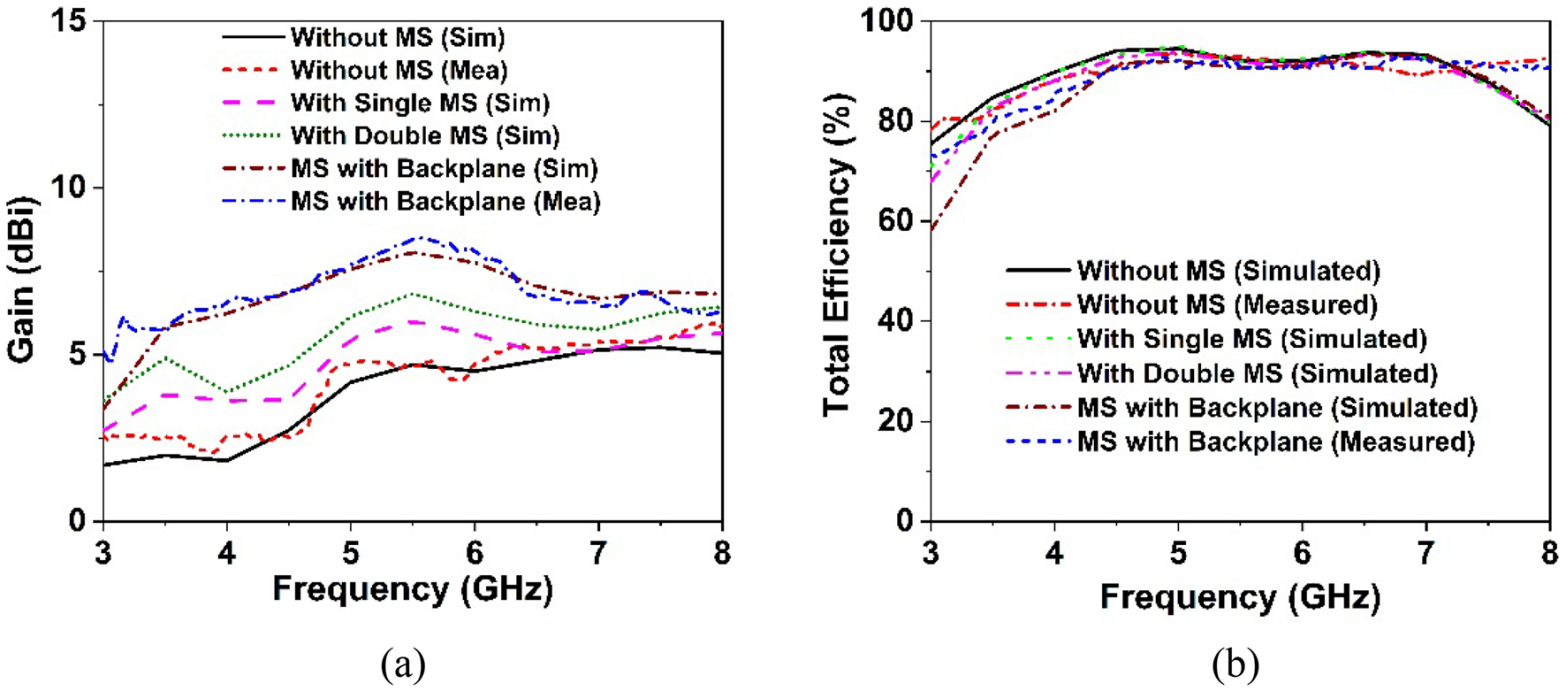
Simulation and measurement outcomes for the suggested antenna with the effect of metasurface (**a**) realized gain and (**b**) total efficiency.

The designed wideband single antenna prototype is fabricated for experimental validation of the proposed design. Figure 7a,b shows images of the proposed single antenna prototype, its parts of construction, and the near-field (SATIMO) measurement setup. To improve antenna performance, the developed metasurface is layered below the antennas, as indicated in Fig. 8a, at the height of h. The 40 mm × 40 mm size of the single and double-layered metasurface has been applied on the backside of the single antenna within the spacing of 12 mm. In addition, a metasurface with a backplane has been placed on the backside of the single antenna at a distance of 12 mm. After applying the metasurface, the single antenna exhibits a substantial increase in performance, as shown in Figs. 8 and 9. Figure 8b shows the plots of the simulated and measured reflection coefficients for a single antenna without and with a metasurface. Notably, the coverage band of the antenna with metasurface is quite similar to that of the antenna without metasurface. Figure 9a,b reveals a comparison of simulated and observed single antenna gain and total efficiency without and with MS over the operating spectrum. As can be seen, the gain of the metasurface antenna is considerably boosted compared to the non-metasurface antenna, increasing from 5.15 to 8 dBi. Single antenna gain is improved by 6 dBi, 6.9 dBi, and 8 dBi for single-layered metasurface, double-layered metasurface, and metasurface with a backplane, respectively. In comparison to other metasurfaces (single- and double-layered MS), the gain of the single antenna with a metasurface that has a copper backplane is the highest of 8 dBi. The metasurface acts as a reflector in this case, reducing the antenna backward radiation and manipulating the EM waves in an in-phase fashion that increases the antenna's radiation efficiency, resulting in gain improvement. The study of the overall efficiency of a single antenna without and with a metasurface is shown in Fig. 9b. Notably, the antenna's efficiency with and without the metasurface is almost identical. In the lower frequency range, the antenna efficiency dropped slightly. The experimental and simulated gain and efficiency curves match well. Although, the simulated and examined outcomes exhibit slight discrepancies because of manufacturing flaws, measurement tolerance, SMA port connection loss, and wire loss. Furthermore, the antenna and MS reflector are sandwiched by a nylon-based spacer, which is another concern that influences the observed outcomes compared to the simulated results.

**Figure 10:**
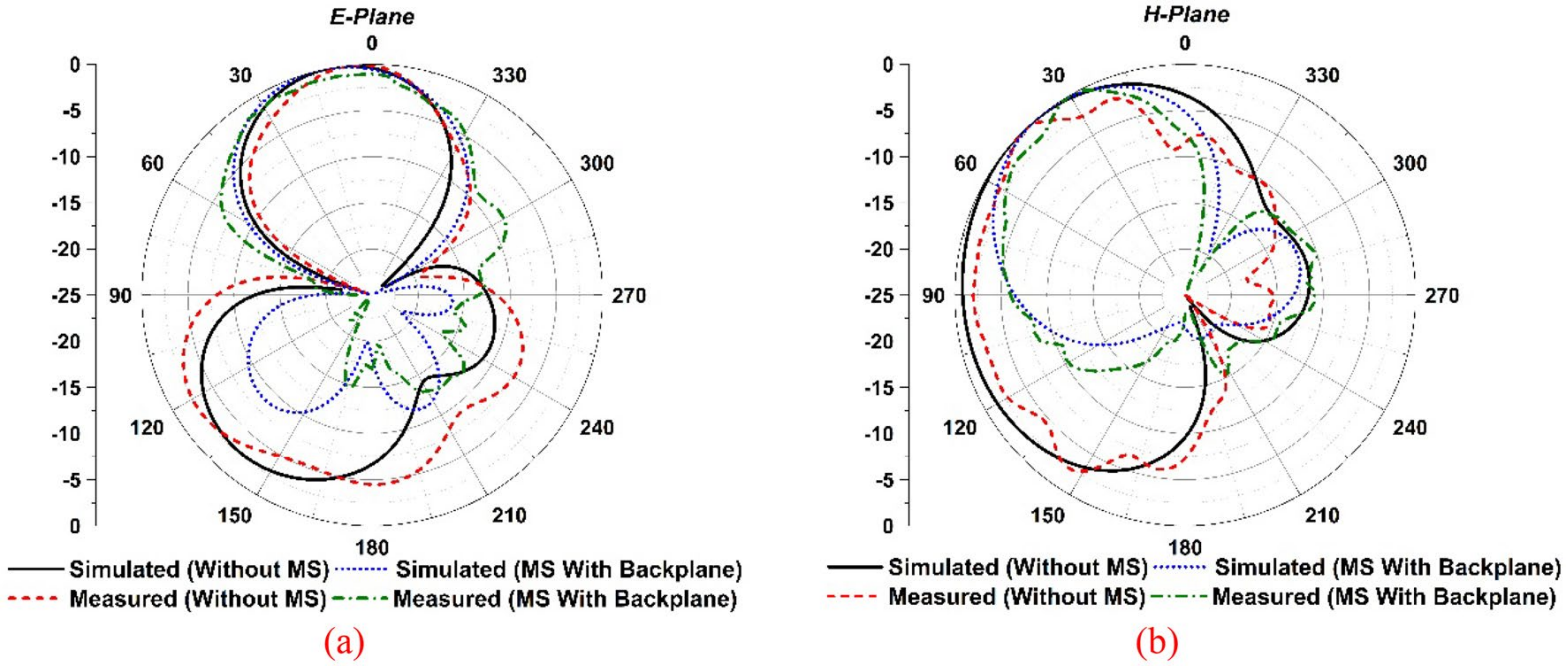
Simulated and examined radiation patterns at 5.5 GHz for developed single antenna without and with MS.

*Radiation pattern Analysis with MS* The single antenna near-field measurements were conducted in the SATIMO near-field experimental environment at the UKM SATIMO near field system laboratory. Figure 10a,b portrays the simulated and observed E- and H-plane radiation patterns at 5.5 GHz for the suggested single antenna with and without MS. The developed single antenna (without MS) offers a consistent bidirectional radiation pattern with side lobe values. After applying the proposed MS reflector, the antenna offers a unidirectional radiation pattern with reducing the back-lobe levels, as shown in Fig. 10a,b. Notably, the proposed single antenna radiation pattern is more stable and unidirectional, with very low back-lobe and side-lobe, when a metasurface with a copper backplane is used. The suggested MM array reflector decreases the antenna’s back and side lobes while improving radiation characteristics that direct current to the unidirectional direction (Fig. 10a,b), resulting in enhanced gain and directivity. It is observed that the experimental radiation pattern is almost comparable to the CST-simulated radiation pattern, with a slight fluctuation due to the misalignment of the different assembly components, measurement tolerance, and cable connection loss. Furthermore, a nylon-based spacer is interposed between the antenna and the MS reflector, which is another concern that influences the observed results compared to the numerical outcomes.

## MIMO antenna design and isolation improvement

**Figure 11:**
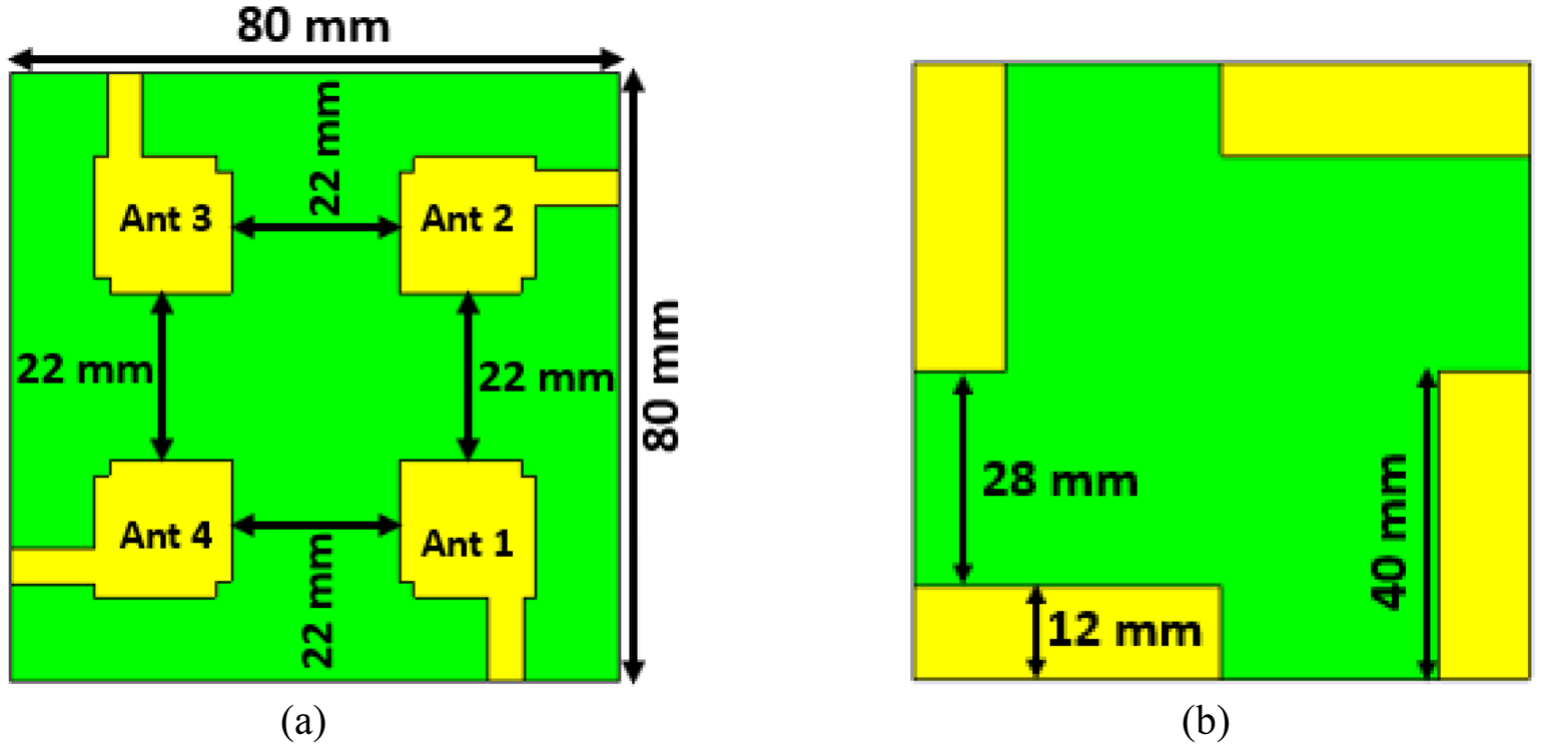
Design mechanism of the proposed MIMO antenna (**a**) top view and (**b**) ground plane. (CST STUDIO SUITE 2019).

**Figure 12:**
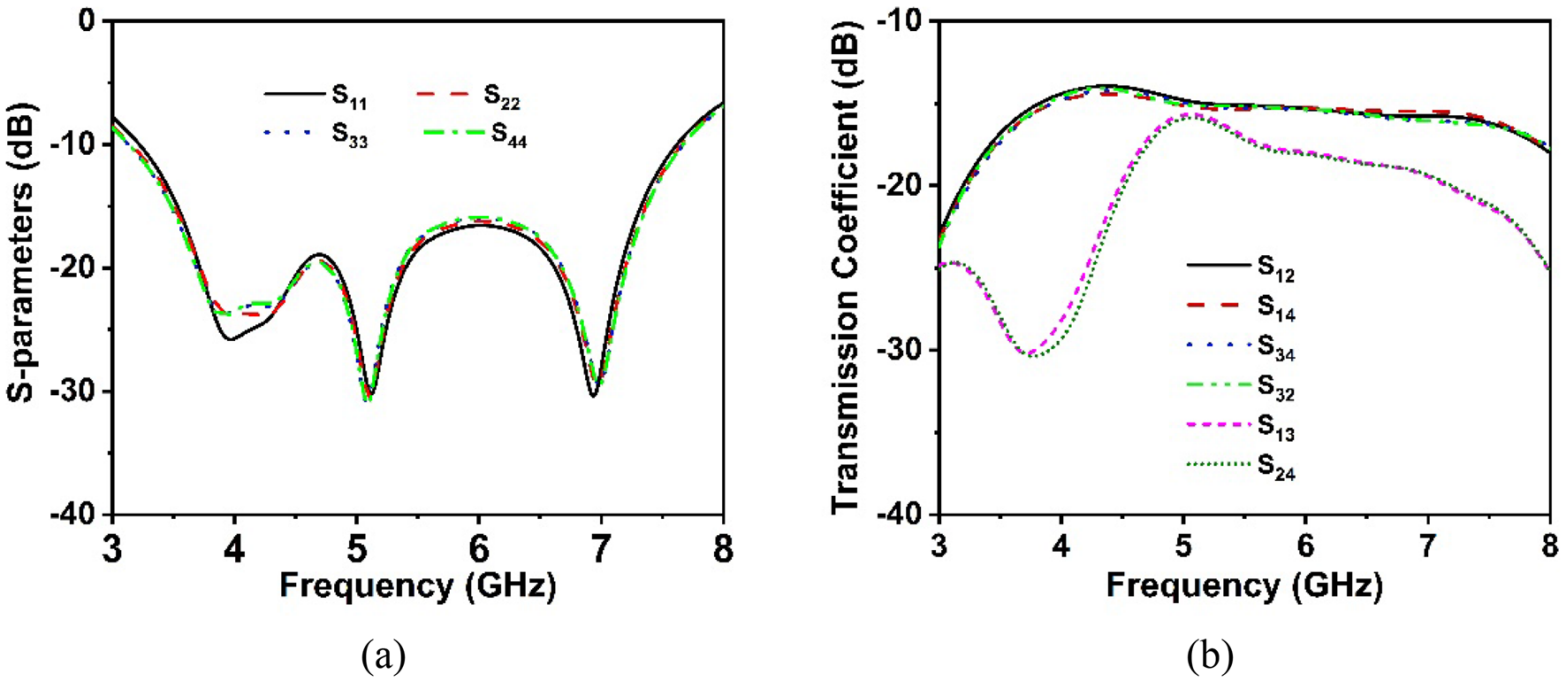
S-parameters results of the MIMO antenna (**a**) reflection coefficient (**b**) transmission coefficient.

The proposed MIMO antenna geometry is represented in Fig. 11, which comprises four single antennas. The four MIMO antenna components are arranged orthogonal to each other on a substrate of 80 mm × 80 mm × 1.575 mm, as indicated in Fig. 11. The interelement distance of the designed MIMO antenna is 22 mm, which is lower than the recently relevant developed MIMO antenna. In addition, the partial ground plane has been positioned in the same manner as the single antenna. The reflection coefficient values for the MIMO antennas (S_11_, S_22_, S_33_, and S_44_) shown in Fig. 12a demonstrate identical behavior to a single element antenna, resonating in the 3.2–7.6 GHz range. Thus, the impedance bandwidth of the MIMO antenna is quite identical to that of a single antenna. The mutual coupling effect between MIMO components is mostly responsible for the MIMO antenna's minor bandwidth loss. The mutual coupling effects on the MIMO components are demonstrated in Fig. 12b, where optimal isolation between the MIMO components has been determined. The least isolation is observed at around − 13.6 dB between antennas 1 and 2, whereas the maximum isolation is found near − 30.4 dB between antennas 1 and 4. Due to the small size and wider bandwidth of this MIMO antenna, it offers low gain and low isolation; thus, gain and isolation enhancements are required.

### MIMO antenna system with MS

**Figure 13:**
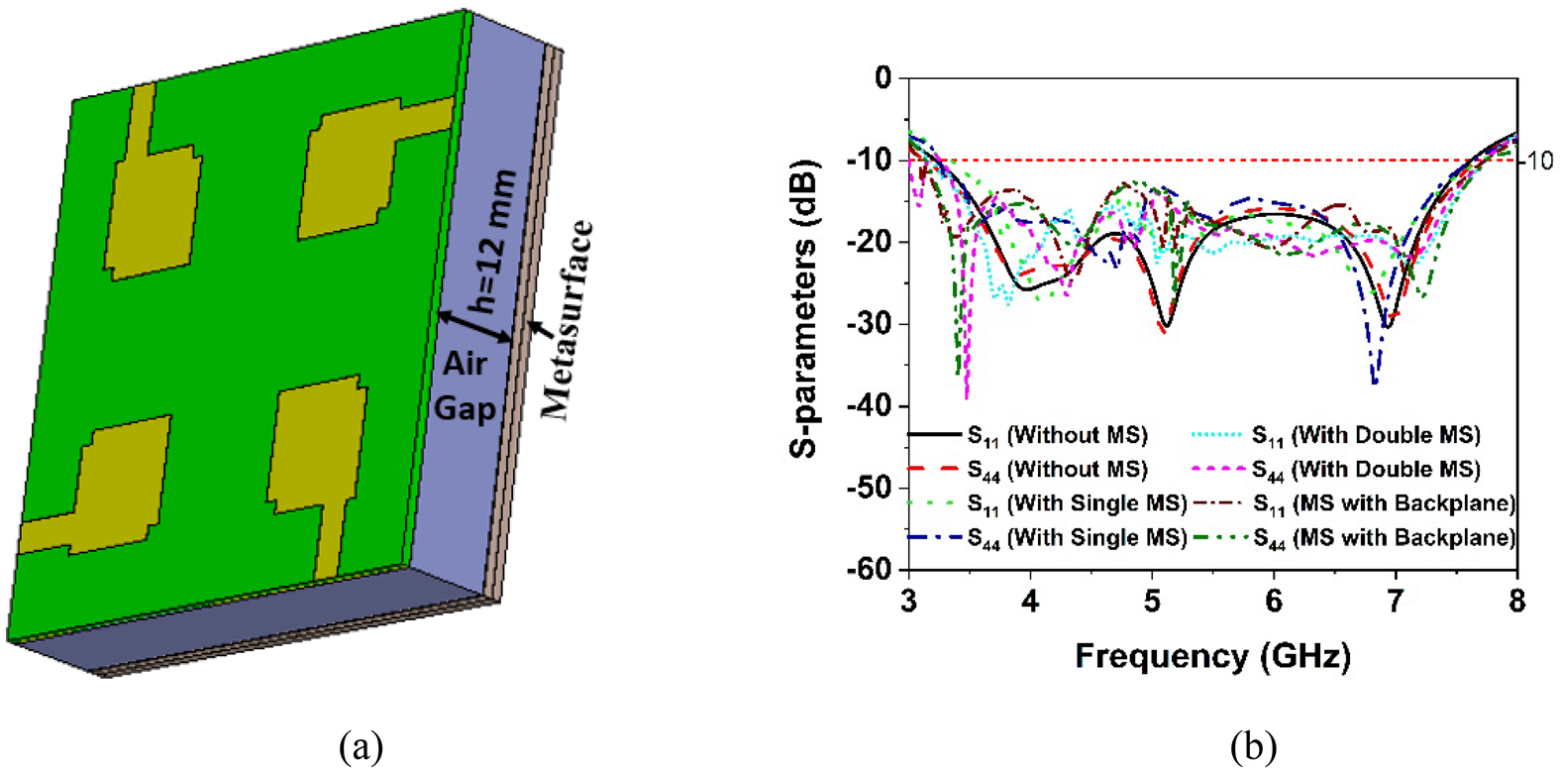
(**a**) CST Simulation setup for suggested MIMO antenna with MS (CST STUDIO SUITE 2019), (**b**) reflection coefficient curves for developed MIMO system without and with MS.

The geometrical arrangement and excitation technique of the suggested metasurface-based MIMO antenna is exhibited in Fig. 13a. 10 × 10 array of MM with dimensions of 80 mm × 80 mm × 1.575 mm has been designed to be used on the rear side of the MIMO antenna at the height of 12 mm, as seen in Fig. 13a. Furthermore, a metasurface with a copper backplane is designed to be applied to a MIMO antenna to improve its performance. The spacing between the metasurface and the MIMO antenna is crucial for achieving high gain while allowing constructive interference between the antenna-generated wave and the metasurface reflected wave. Numerous simulations are run to optimize the height between the antenna and the metasurface while maintaining the quarter wavelength standard to obtain the highest gain and isolation between MIMO elements. The considerable enhancements in MIMO antenna performance achieved by employing a metasurface with a backplane over a metasurface without a backplane are given in the subsequent sections.

### MIMO antenna performance analysis

#### Reflection and transmission coefficient analysis

**Figure 14:**
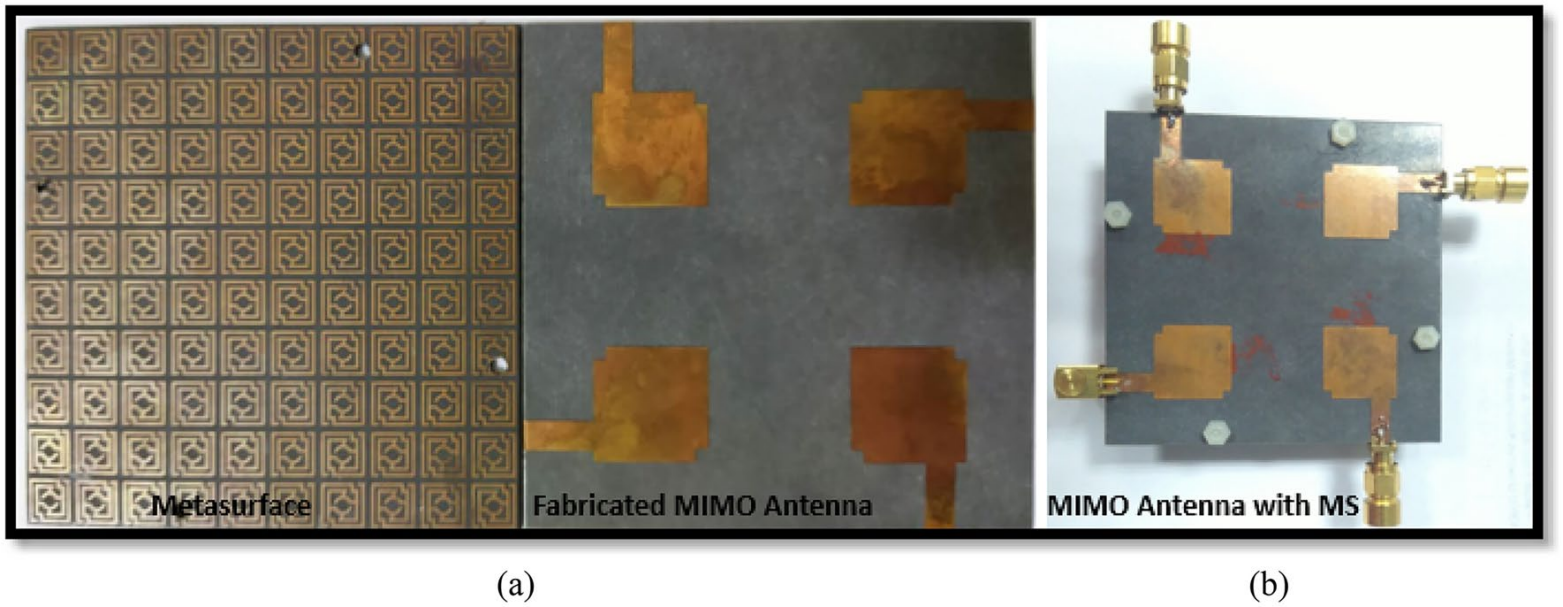
(**a**) Fabricated MIMO antenna prototype with various parts of assembly (**b**) developed MIMO system.

**Figure 15:**
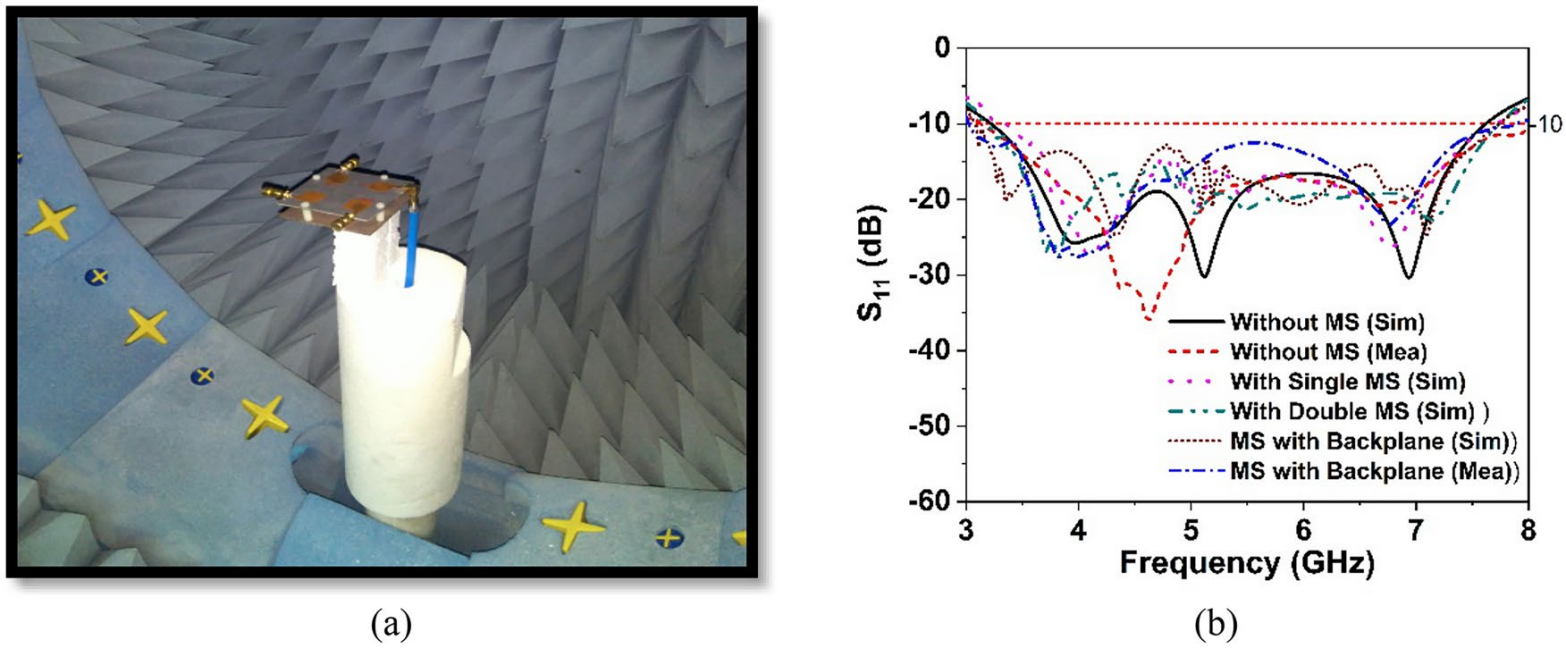
(**a**) SATIMO near-field measurement photograph (**b**) simulation and experimental S_11_ curves for MIMO antenna with and without MS.

The reflection coefficients of a MIMO antenna with and without a metasurface are displayed in Fig. 13b, where S_11_ and S_44_ are presented due to the almost identical behavior of all antennas in the MIMO system. Notably, the − 10 dB impedance bandwidth of the MIMO antenna without and with a single metasurface is nearly identical. In contrast, the impedance bandwidth of the suggested MIMO antenna is improved with a double-layered MS and MS with a backplane. It is noted that, without MS, the MIMO antenna provides 81.5% fractional bandwidth (3.2–7.6 GHz) relative to the central frequency. The integration of MS with a backplane improved the impedance bandwidth of the suggested MIMO antenna to 86.3% (3.08–7.75 GHz). Although the double-layered MS improves bandwidth, the improvement is less than that of the MS with a copper backplane. Moreover, the double-layered MS enlarges the antenna, which increases the cost and limits the application area. The designed MIMO antenna and metasurface reflector are fabricated and examined in order to verify the simulation results and assess real-world performance. Figure 14a depicts the fabricated MS layer and MIMO antenna with various assembly components, whereas Fig. 14b shows photos of the developed MIMO system. Four nylon spacers are utilized to stack the MIMO antenna on top of the metasurface, as indicated in Fig. 14b. Figure 15a presents a snapshot of the near-field experimental setup for a developed MIMO antenna system. The PNA network analyzer (Agilent Technologies PNA N5227A) was used to assess the scattering parameters, and the near-field radiation performance was evaluated and characterized at the UKM SATIMO near-field system lab.

This section presents a comparative study of the simulated and observed S-parameters for the suggested 5G MIMO antenna. The experimental reflection coefficient plots for the MS integrated 4-element MIMO antenna are given in Fig. 15b in contrast to the CST simulated findings. The experimental reflection coefficients are found to be identical to the CST computed results, with a little difference owing to manufacturing defects and experimental tolerances. Furthermore, the observed reflection coefficient of the proposed MS-based MIMO prototype encompasses the 5G sub-6 GHz spectrum with a wide impedance bandwidth of 4.8 GHz, implying that 5G applications are conceivable. Although, the measured resonance frequencies, bandwidth, and amplitude deviated slightly from the CST simulated findings. Fabrication defects, coaxial cable and SMA connection loss, and open-air measurement arrangement contribute to the discrepancies between measured and simulated results. However, despite these shortcomings, the proposed MIMO performs well, with strong accordance between simulation and measurement, making it perfect for 5G sub-6 GHz wireless applications.

**Figure 16:**
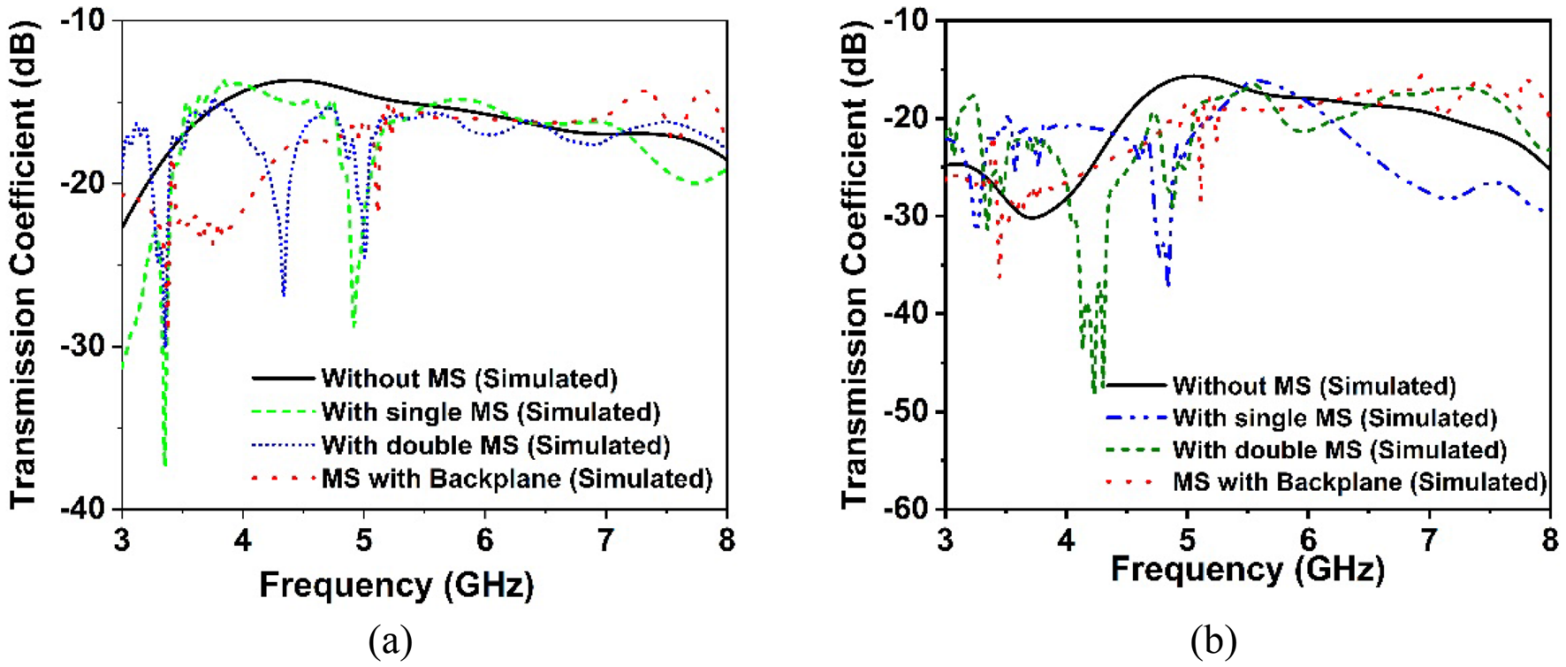
Simulated isolation curves for MIMO elements without and with the MS layer: (**a**) S_12_, S_14_, S_34_ and S_32_ and (**b**) S_13_ and S_24_.

**Figure 17:**
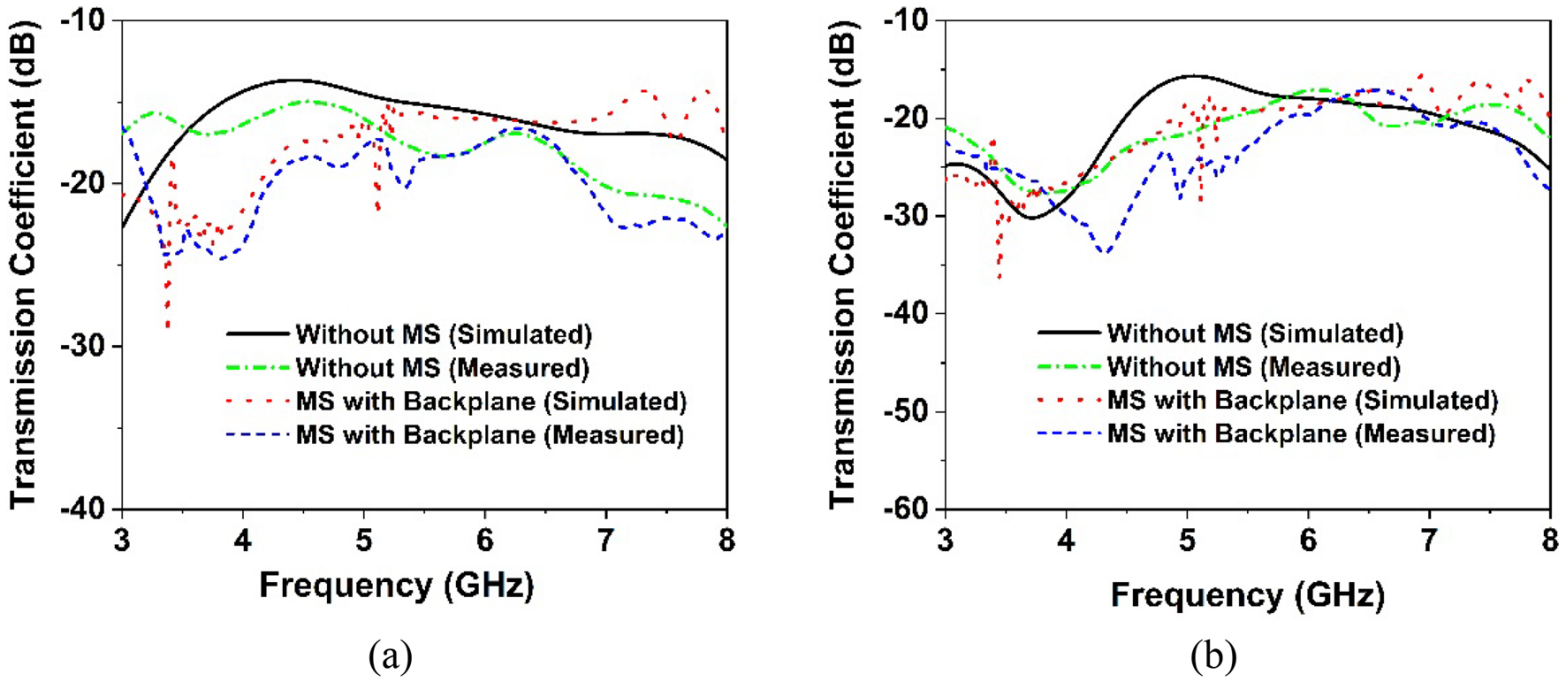
Experimental transmission coefficient curves of the proposed without and with MS-based MIMO antenna: (**a**) S_12_, S_14_, S_34_ and S_32_ and (**b**) S_13_ and S_24_.

The simulated and observed transmission coefficient curve of the MIMO antenna is illustrated in Figs. 16a,b and 17a,b, respectively, demonstrating the mutual interaction of the MIMO components. The isolation between the MIMO antennas improves noticeably when the metasurface is applied to the MIMO antenna. The isolation plots between the nearby antenna elements, S_12_, S_14_, S_23_, and S_34_ show similar curves, whereas the diagonal MIMO antennas, S_13_ and S_42_, exhibit identical high isolation due to the larger distance between them. The simulated transmission characteristics of the adjacent antennas are described in Fig. 16a. It is noteworthy to mention that the minimum isolation for a MIMO antenna without a metasurface is − 13.6 dB and the metasurface with a backplane is − 15.5 dB over the 5G sub-6 GHz operating spectrum. The transmission coefficient graphs (Fig. 16a) demonstrate that the metasurface with a backplane significantly improved isolation between MIMO antenna elements compared to single- and double-layered metasurfaces. On neighboring antenna elements, the single and double-layered metasurface gives minimum isolation of approximately − 13.68 dB and − 14.78 dB, whereas the metasurface with a copper backplane provides approximately − 15.5 dB.

The transmission coefficient plots of MIMO diagonal antennas before and later adding the MS layer are illustrated in Fig. 16b. Notably, the minimum isolation between diagonal antennas (antennas 1 and 3) without a metasurface is − 15.6 dB at the working spectrum, whereas the metasurface with a backplane is − 18 dB. The metasurface approach greatly diminishes the mutual coupling impact between the MIMO diagonal antennas. The maximum isolation for a single-layered metasurface is − 37 dB, whereas this value drops to − 47 dB for a double-layered metasurface. The maximum isolation for a metasurface with a copper backplane is − 36.2 dB, which decreases as the frequency range increases. In comparison to single and double-layered metasurfaces without a backplane, a metasurface with a backplane provides excellent isolation throughout the desired operating band, especially at the 5G sub-6 GHz frequency band as seen in Fig. 16a,b. At the most popular and widely used 5G sub-6 GHz band (3.5 GHz), isolation between MIMO components is lower (almost near to without MS) for single- and double-layered metasurfaces than metasurface with a copper backplane (see Fig. 16a,b). The measured transmission coefficient findings are presented in Fig. 17a,b, displaying isolation for nearby antennas (S_12_, S_14_, S_34_, and S_32_) and diagonal antennas (S_24_ and S_13_) respectively. From these figures (Fig. 17a,b), it can be observed that the experimental isolation between MIMO components is in excellent agreement with the simulated isolation. Although, due to manufacturing flaws, SMA port connection, and wire loss, the CST simulated, and measured values exhibit minor discrepancies. Furthermore, the antenna and MS reflector are sandwiched by a nylon-based spacer, which is another concern that influences the observed outcomes compared to the simulated results.

**Figure 18:**
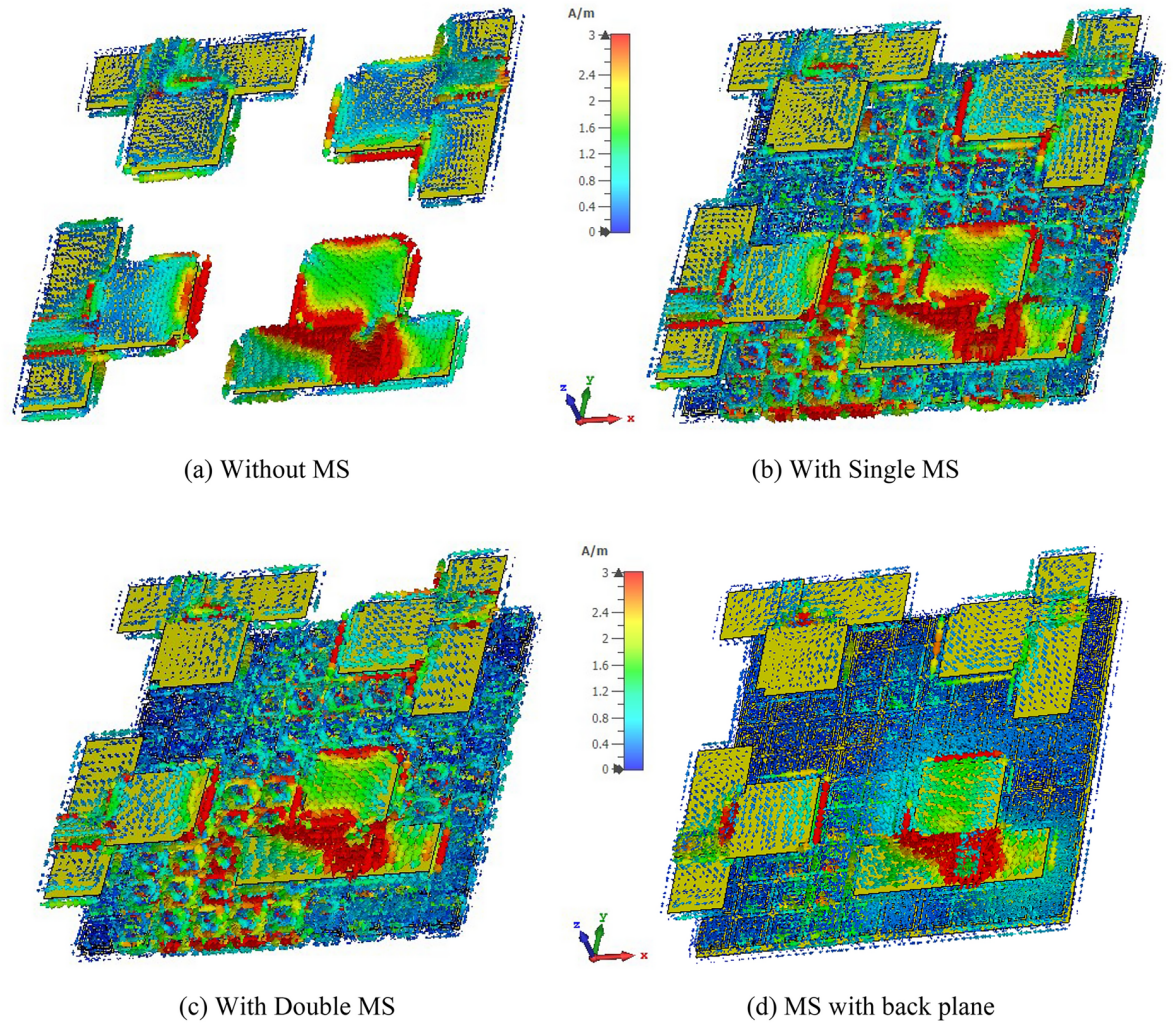
The surface current pattern of the suggested MIMO antenna at 5.5 GHz (**a**) Without MS, (**b**) Single-layered MS, (**c**) Double-layered MS and (**d**) Single-layered MS with a copper backplane. (CST STUDIO SUITE 2019).

The surface current distribution is studied at 5.5 GHz to rationalize the involvement of the metasurface in mutual coupling reduction via surface wave suppression^42^. The surface current distribution of the suggested MIMO antenna is shown in Fig. 18, where antenna 1 is excited, and the rest of the antenna is terminated by a 50-Ω load. When antenna 1 is stimulated, a significant mutual coupling current appears at the neighboring antenna at 5.5 GHz without the presence of a metasurface, as presented in Fig. 18a. Conversely, by applying the metasurface as indicated in Fig. 18b–d, the isolation between adjacent antennas improves. It is noticed that the influence of adjacent field mutual coupling is minimized by dispersing the couple current to the MS layer traversing antiparallel direction in neighboring rings of the unit cell and adjacent unit cells of the MS. The distributed antenna coupling current to the unit cells of the MS is the key approach for enhancing isolation between MIMO components. Thus, the coupling current between the MIMO components is reduced greatly, while isolation is significantly improved. Since the coupled field is widely distributed in the unit cells, the metasurface with a copper backplane isolates the MIMO antenna components noticeably than the single and double layer metasurfaces (Fig. 18d). Moreover, the developed MIMO antenna offers very low back and side propagation, introducing the unidirectional radiation pattern, hence enhancing the gain of the suggested MIMO antenna.

#### MIMO gain and total efficiency analysis

**Figure 19:**
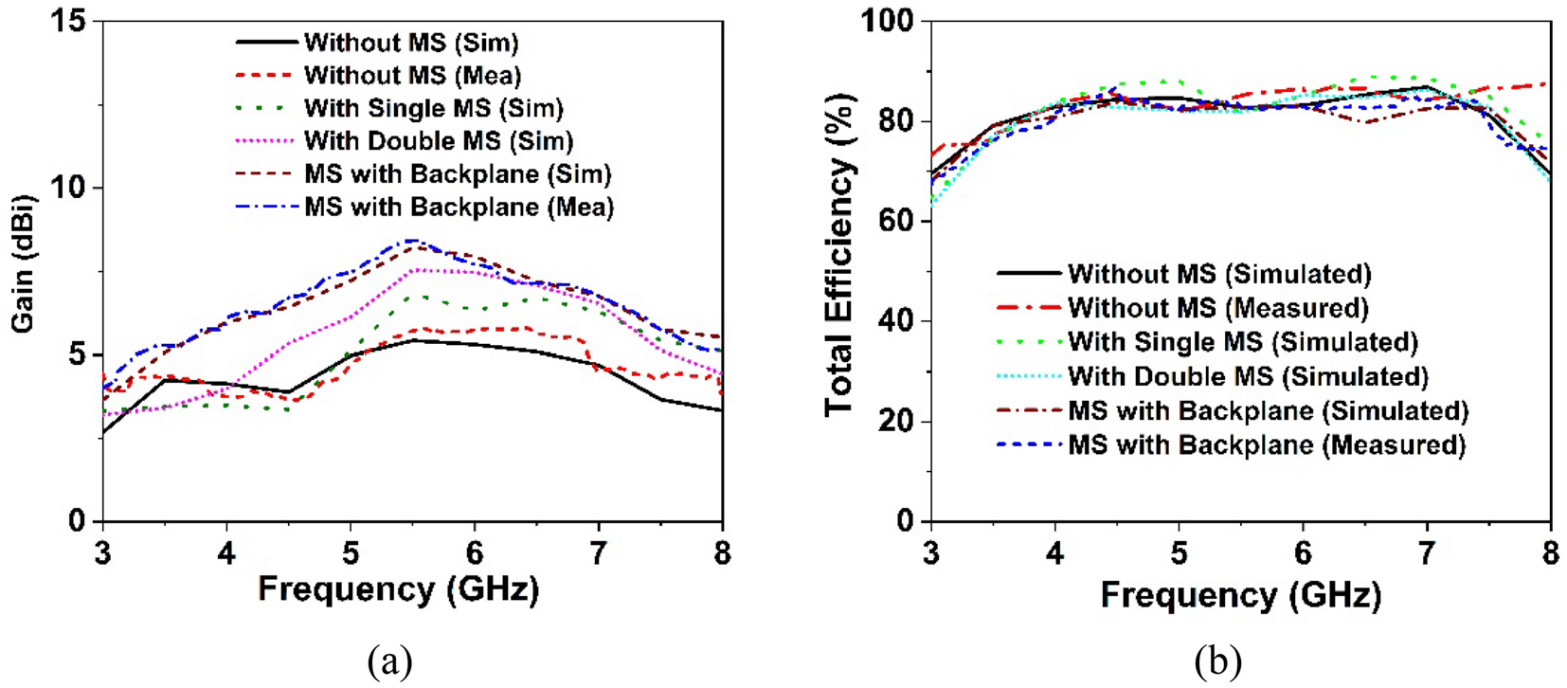
Simulation and experimental results of suggested MIMO antenna with the effect of metasurface (**a**) realized gain and (**b**) total efficiency.

Within the operational frequency, Fig. 19a exhibits the simulated and observed realized gain of the developed MIMO antenna without and with a metasurface. The simulated realized gain of a MIMO antenna without the metasurface is 5.4 dBi, which is indicated in Fig. 19a. Because of the mutual coupling effect between MIMO components, the realized gain of the suggested MIMO antenna is indeed 0.25 dBi higher than that of the single antenna. The inclusion of the metasurface results in significant gain and isolation between MIMO components. Thus, the suggested MIMO antenna with a metasurface offers a high realized gain of up to 8.3 dBi. As seen in Fig. 19a, the gain is enhanced by 1.4 dBi, when a single metasurface is used on the rear of the MIMO antenna. Gain is enhanced by 2.1 dBi upon doubling the metasurface, as seen in Fig. 19a. However, the anticipated maximal realized gain of 8.3 dBi is obtained by employing a metasurface with a copper backplane. Notably, the single- and double-layered metasurfaces have maximum realized gains of 6.8 dBi and 7.5 dBi, respectively, while the metasurface with a backplane has a maximum realized gain of 8.3 dBi. The metasurface layer on the antenna's backside works as a reflector, reflecting the antenna backside radiation and enhancing the developed MIMO antenna’s front-to-back (F/B) ratio. Moreover, the high-impedance MS reflector manipulates electromagnetic waves in an in-phase fashion, resulting in additional resonances and improving the radiation characteristics of the suggested MIMO antenna. The MS reflector, which is fitted behind the MIMO antenna, is observed to significantly enhance the realized gain, which is verified by the experimental results. The observed and simulated realized gain of the developed MIMO antenna prototype is almost identical; nevertheless, the measured gain is greater than the simulated gain at some frequencies, particularly for MIMO without MS. This fluctuation in experimental gain is driven by the measurement tolerance, cable loss and coupling of the nylon spacer in the antenna system. The MIMO antenna without the metasurface offers a peak measured gain of 5.8 dBi, whereas the metasurface with a copper backplane is 8.5 dBi. It is notable to mention that the proposed complete 4-port MIMO antenna system with MS reflector exhibits a high realized gain in the experimental and numerical conditions.

The total efficiency of the suggested MIMO system without and with a metasurface reflector is depicted in Fig. 19b. The minimum efficiency after employing MS with a backplane is determined to be more than 73% (up to 84%) in Fig. 19b. The total efficiency of the developed MIMO antenna without and with MS is determined to be almost the same, with minor differences noted as compared to the simulated values. This could happen because of measurement tolerance as well as the spacer that is employed between the antenna and the MS reflector, among other reasons. The measured realized gain and total efficiency at the entire frequency are nearly similar to the simulated results, showing that the suggested MIMO prototype performs as expected and that the recommended MS-based MIMO antenna is suitable for 5G communications. Due to the error involved in the experimental investigation, the overall laboratory experimental results differ from the simulated findings. The proposed prototype performance is influenced by an impedance mismatch between the antenna and the SMA connector, coaxial cable connections loss, the soldering effect, and the proximity of various electronic equipment to the experimental setup.

**Figure 20:**
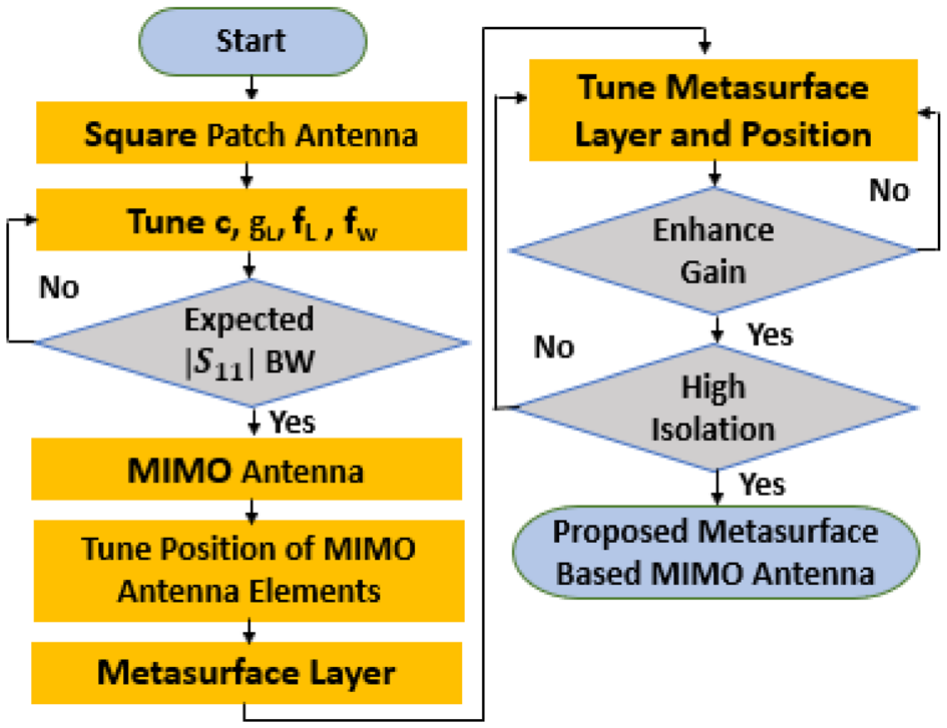
The flowchart for designing and optimizing the proposed MS-based 4-port MIMO antenna.

The mentioned antenna design progression and optimization process are depicted in Fig. 20 via a flowchart. In this flowchart, the proposed MIMO antenna design principle and the parameters that play a key role in optimizing the antenna are described orderly to reach the desired high gain and high isolation within the wide operating frequency.

#### MIMO radiation pattern analysis with MS

**Figure 21:**
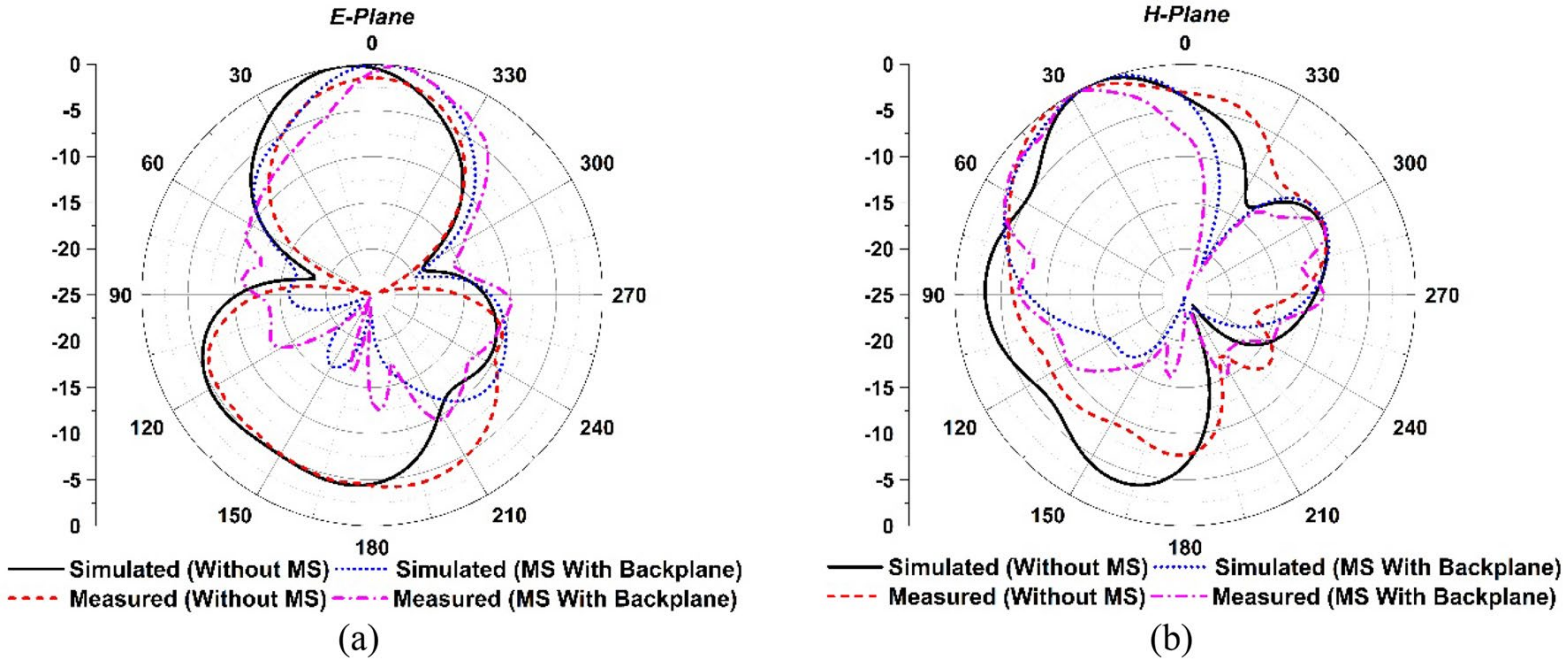
Simulated and examined radiation patterns at 5.5 GHz for developed MIMO antenna without and with MS.

The MIMO antenna near-field findings have been measured in the SATIMO near-field experimental environment at the SATIMO near field system lab, UKM. Figure 21a,b depicts the simulated and observed E- and H-plane radiation patterns for the reported MIMO antenna with and without MS at a 5.5 GHz operating frequency. At 5.5 GHz in the operational band, the developed MIMO antenna without MS provides a consistent bidirectional radiation pattern with side lobe values. After applying the MS reflector, the antenna offers a unidirectional radiation pattern with reducing the back-lobe levels, as shown in Fig. 21a,b. Notably, the suggested MIMO antenna radiation pattern is more stable and unidirectional with very low back-lobe and side-lobe when employing a metasurface with a copper backplane than without MS. The proposed MM array reflector reduces the antenna’s back and side lobes while also improving radiation properties that direct current to the unidirectional direction (Fig. 21a,b), resulting in enhanced gain and directivity. The measured radiation pattern has been obtained from port 1, while the 50-Ω load terminated the remaining ports. It is observed that the experimental radiation pattern is almost identical to the CST simulated radiation pattern, although a little variation is owing to the misalignment of the various parts of the assembly, terminated port reflection and cable connection loss. Furthermore, a nylon-based spacer is interposed between the antenna and the MS reflector, which is another concern that influences the observed findings compared to the predicted results.

#### MIMO diversity performance

It is vital to note that port isolation and correlation characteristics should be required to assess the performance of a MIMO system. The diversity performance of the suggested MIMO system, including envelope correlation coefficient (ECC) and diversity gain (DG), has been explored to illustrate the durability of the developed MIMO antenna system. The ECC and DG of MIMO antennas can be utilized to assess their performance, as they are important aspects of MIMO system performance. The next sections go through these features in detail for the suggested MIMO antenna.

*Envelope Correlation Coefficient (ECC)* When considering any MIMO system, the ECC determines how well the constituent elements correlate with one another in terms of their particular properties. Thus, the ECC exhibits how well the channel isolation in the wireless communication network is. ECC (Envelope Correlation Coefficient) of the developed MIMO system can be determined from the S-Parameters and the far-field radiation. From Eqs. (7) and (8), it is possible to determine the ECC of the suggested MIMO antenna^31^.

7 $$\rho_{eij} = \frac{{\left| {S_{ii} *S_{ij} + S_{ji} *S_{jj} } \right|^{2} }}{{\left( {1 - \left| {S_{ii} } \right|^{2} - S_{ij}^{2} } \right)\left( {1 - \left| {S_{ji} } \right|^{2} - S_{jj}^{2} } \right)}}$$

8 $$\rho_{eij} = \frac{{\left| {\int {\int_{0}^{4\pi } {\left[ {\vec {R_{i}} \left( {\theta ,\varphi } \right) \times \vec{R}_{j} \left( {\theta ,\varphi } \right)} \right] d\Omega } } } \right|^{2} }}{{\int {\int_{0}^{4\pi } {\left| {\vec{R_{i} } \left( {\theta ,\varphi } \right)} \right|^{2} d\Omega \int {\int_{0}^{4\pi } {\left| {\vec{R}_{j} \left( {\theta ,\varphi } \right)} \right|^{2} d\Omega } } } } }}$$

**Figure 22:**
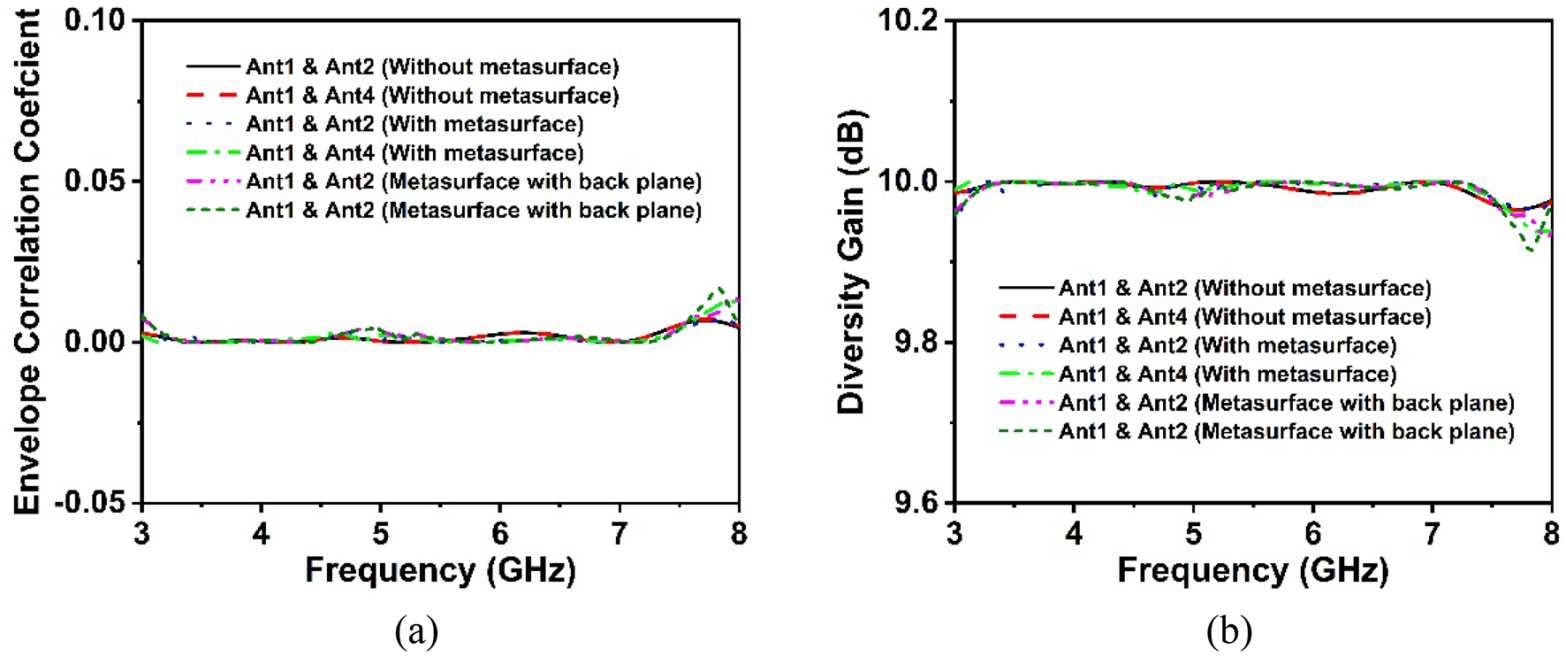
Diversity features of the designed MIMO antenna (**a**) ECC and (**b**) DG.

The reflection coefficient is denoted by *S*_*ii*_, while *S*_*ij*_ denotes the transmission coefficient. The three-dimensional radiation patterns of the *j*th and *i*th antennas are represented by $\vec{R}_{j} \left( {\theta ,\varphi } \right)$ and $\vec{{R_{i}}} \left( {\theta ,\varphi } \right)$ as well as the solid angle represented by ${\Omega }$. The ECC curve of the suggested antenna is represented in Fig. 22a, with a value of less than 0.004, which is significantly lower than the allowed value of 0.5 for wireless systems. Therefore, the reduced ECC value implies that the suggested 4-port MIMO system provides an excellent diversity pattern^43^.

*Diversity Gain (DG)* The DG is another MIMO system performance metric that describes how the diversity scheme affects the radiated power. The relationship expression (9) determines the DG of a developed MIMO antenna system, as mentioned in^31^.

9 $${\text{Diversity}}\;{\text{Gain}} = 10\sqrt {1 - \left| {\rho_{eij} } \right|^{2} }$$

Figure 22b shows the DG plot of the suggested MIMO system, where the DG value is very near to 10 dB. The designed MIMO system has a DG value of greater than 9.98 dB across all antennas.

## Comparative analysis of similar work

Table 1 compares the proposed metasurface-based MIMO antenna  with recently developed similar MIMO systems. The comparison took into account various performance parameters, including bandwidth, gain, maximum isolation, total efficiency and diversity performance. Researchers in^5,44–47^ introduced a variety of MIMO antenna prototypes with gain and isolation improvement techniques. In contrast to earlier reported works, the suggested MIMO system with a metasurface reflector outperforms them in the field of bandwidth, gain and isolation. Moreover, the developed MIMO system exhibits excellent diversity performance and total efficiency with miniaturized dimension than related reported antennas. Although the antennas reported in^5,46^ offers higher isolation than our suggested antenna, however, these antennas suffer from large dimension, low gain, narrow bandwidth, and poor MIMO performance. The 4-port MIMO antenna presented in^45^ demonstrated high gain and efficiency, but this design offers low isolation, large dimension and poor diversity performance. On the other side, the small size antenna system is suggested in^47^ with very low gain and operating bandwidth, whereas our proposed 4-port MS-based MIMO system exhibits a small dimension with high gain, high isolation and better MIMO performance. Thus, the proposed metasurface-based MIMO antenna might be the dominant contender for the sub-6 GHz 5G communication system.

**Table 1:** Proposed MIMO system performance comparison with relevant work.

Ref	Size (mm^2^)	Antenna type	Bandwidth (GHz)	Max. gain (dBi)	Total efficiency	Min. isolation (dB)	Max. isolation (dB)	ECC (dB)	DG (dB)
^44^	75 × 150	MIMO + Parasitic structure	3.4–3.8	6.5	60–70%	≥ 15	29	< 0.01	Not given
^5^	150 × 70	MIMO + Neutral line	3.1–3.85 4.8–6	Not given	60–75%	≥ 17	24	< 0.06	Not given
^45^	129.5 × 129.5	MIMO + cavity-backed	1.55–6	10	80–84%	≥ 15	36	< 0.05	Not given
^46^	150 × 150	Annular-ring patch	3.3–5	6.8	84–92%	≥ 16.5	26	< 0.05	Not given
^47^	26 × 26	MIMO (slot antenna)	5.6–5.8	1.7	Not given	≥ 15.4	32	< 0.01	Not given
Prop. work	80 × 80	MIMO + MS	3.08–7.75	8.3	73–84%	≥ 15.5	36.2	< 0.004	> 9.98

## Conclusion

A metasurface reflector-based four-port wideband MIMO antenna with high gain and isolation is presented, supporting 5G sub-6 GHz applications. A microstrip line feeds a square radiating patch, which is truncated by a square at the diagonal corners. The proposed MS and antenna radiator is realized on the similar substrate material of Rogers RT5880 to achieve excellent performance in the 5G high-speed communication systems. This proposed MIMO antenna is distinguished by its wide coverage and high gain while also providing sound isolation between MIMO components and excellent efficiency. The developed single antenna has a miniaturized size of 0.58λ × 0.58λ × 0.02λ with a 5 × 5 metasurface array, offering a 4.56 GHz wide operational bandwidth, 8 dBi peak gain and excellent efficiency that is confirmed by the measured results. The suggested four-port MIMO antenna (2 × 2 array) is developed with a dimension of 1.05λ × 1.05λ × 0.02λ by aligning each proposed single antenna orthogonally to the other. The 10 × 10 array of suggested MM is assembled beneath the MIMO antenna with a height of 12 mm, which reduces the backward radiation and decreases mutual coupling between MIMO components, thus enhancing gain and isolation. The experimental and simulated findings reveal that the developed MIMO prototype operates over a wide frequency spectrum of 3.08–7.75 GHz, covering the 5G sub-6 GHz spectrum. Furthermore, the suggested MS-based MIMO antenna improves its gain by 2.9 dBi, reaching a maximum gain of 8.3 dBi, and provides excellent isolation between MIMO components (> 15.5 dB), confirming MS's contribution. Besides that, the suggested MIMO antenna provides a high average total efficiency of 82% and a short interelement distance of 22 mm. The antenna demonstrated outstanding MIMO diversity characteristics, including a very high DG (over 9.98 dB), a very little ECC (less than 0.004), and a unidirectional radiation pattern. The measured results resembled the simulated outcomes well. These properties validate that the developed four-port MIMO antenna system could be a feasible choice for 5G sub-6 GHz communication systems.
