# Author Correction: Gain and isolation enhancement of a wideband MIMO antenna using metasurface for 5G sub-6 GHz communication systems

**DOI:** 10.1038/s41598-023-29750-2

**Published:** 2023-02-16

**Authors:** Md. Mhedi Hasan, Mohammad Tariqul Islam, Md Samsuzzaman, Mohd Hafiz Baharuddin, Mohamed S. Soliman, Ahmed Alzamil, Iman I. M. Abu Sulayman, Md. Shabiul Islam

**Affiliations:** 1 Department of Electrical, Electronic and Systems Engineering, Faculty of Engineering and Built Environment, Universiti Kebangsaan Malaysia, 43600 Bangi, Malaysia; 2 Department of Information and Communication Technology (ICT), Faculty of Engineering, Comilla University, Cumilla, 3506 Bangladesh; 3 Electrical Engineering Department, College of Engineering, University of Ha’il, Ha’il, 81481 Saudi Arabia; 4 Department of Computer and Communication Engineering, Faculty of Computer Science and Engineering, Patuakhali Science and Technology University, Patuakhali, Bangladesh; 5 Department of Electrical Engineering, College of Engineering, Taif University, PO Box 11099, Taif, 21944 Saudi Arabia; 6 Faculty of Engineering (FOE), Multimedia University, Persiaran Multimedia, 63100 Cyberjaya, Selangor Malaysia

Correction to: *Scientific Reports* 10.1038/s41598-022-13522-5, published online 08 June 2022

The original version of this Article omitted an affiliation for Md. Mhedi Hasan. The correct affiliations are listed below.

Department of Electrical, Electronic and Systems Engineering, Faculty of Engineering and Built Environment, Universiti Kebangsaan Malaysia, 43600 Bangi, Malaysia.

Department of Information and Communication Technology (ICT), Faculty of Engineering, Comilla University, Cumilla 3506, Bangladesh.

The original Article has been corrected.

